# Constrained thermoresponsive polymers – new insights into fundamentals and applications

**DOI:** 10.3762/bjoc.17.138

**Published:** 2021-08-20

**Authors:** Patricia Flemming, Alexander S Münch, Andreas Fery, Petra Uhlmann

**Affiliations:** 1 Leibniz-Institut für Polymerforschung Dresden e.V., Hohe Straße 6, 01069 Dresden, Germany; 2 Technische Universität Dresden, 01062 Dresden, Germany; 3 University of Nebraska-Lincoln, NE 68588, Lincoln, USA

**Keywords:** lower critical solution temperature (LCST), responsive coating, smart material, thermoresponsive polymer, upper critical solution temperature (UCST)

## Abstract

In the last decades, numerous stimuli-responsive polymers have been developed and investigated regarding their switching properties. In particular, thermoresponsive polymers, which form a miscibility gap with the ambient solvent with a lower or upper critical demixing point depending on the temperature, have been intensively studied in solution. For the application of such polymers in novel sensors, drug delivery systems or as multifunctional coatings, they typically have to be transferred into specific arrangements, such as micelles, polymer films or grafted nanoparticles. However, it turns out that the thermodynamic concept for the phase transition of free polymer chains fails, when thermoresponsive polymers are assembled into such sterically confined architectures. Whereas many published studies focus on synthetic aspects as well as individual applications of thermoresponsive polymers, the underlying structure–property relationships governing the thermoresponse of sterically constrained assemblies, are still poorly understood. Furthermore, the clear majority of publications deals with polymers that exhibit a lower critical solution temperature (LCST) behavior, with PNIPAAM as their main representative. In contrast, for polymer arrangements with an upper critical solution temperature (UCST), there is only limited knowledge about preparation, application and precise physical understanding of the phase transition. This review article provides an overview about the current knowledge of thermoresponsive polymers with limited mobility focusing on UCST behavior and the possibilities for influencing their thermoresponsive switching characteristics. It comprises star polymers, micelles as well as polymer chains grafted to flat substrates and particulate inorganic surfaces. The elaboration of the physicochemical interplay between the architecture of the polymer assembly and the resulting thermoresponsive switching behavior will be in the foreground of this consideration.

## Introduction

During the last decades, the class of stimuli-responsive materials has entered the focus of scientific research and applied polymer science [1–9]. They are characterized primarily by their ability to adapt spontaneously and reversibly to changes of environmental conditions because of their characteristic physical and chemical properties. For this reason, they are also referred to as "smart materials". Due to their very flexibly designable organic structure, the physicochemical properties of polymers can be adjusted precisely, making them ideal chemical components for the generation of smart devices. Depending on the polymer structure, including backbone and functional groups [10], their properties can be reversibly influenced by external chemical (e.g., pH value [11–12], ionic strength [13], polarity of solvent [14]), biological (e.g., bacteria, biomolecules [15], enzymes [16]) or physical stimuli (e.g., light [17–19], temperature [12,18], mechanical forces, as well as electric [20–21] and magnetic fields [22–24]) [1,25–26]. As a result of these environmental triggers, smart materials exhibit a defined reversible change in their physicochemical properties (e.g., solubility, viscosity) and can respond to stimuli in several ways by altering light transmitting abilities, shape, color, conductivity, as well as wettability [27–28]. Therefore, such functional polymers have a huge potential in numerous areas of application including biomedicine [29–30], drug delivery [12,31], tissue engineering [32–33], analytic application [18], environmental applications [34–35], sensors [36–38], actuators [37–39] and other applications [5].

For many of these purposes, it is essential to tether such polymers to interfaces or arrange them into confined polymeric architectures, such as star polymers or micelles, in a defined way. All these arrangements have in common that the mobility, i.e., the degrees of freedom of movement of the polymer chains, is limited compared to free polymers in solution, which affects the responsiveness of the polymers. The switching behavior can thus be controlled not only by the chemical composition of the polymer and its chain length, but also by its specific arrangement, for example by varying the grafting density [40–42]. Therefore, a transfer of the physicochemical concepts valid for free polymer chains in solution is only conditionally applicable to constrained polymer topologies. The most commonly used stimulus to induce responsive behavior is the temperature [2,43–45]. The physicochemical basis for this is a temperature-dependent solubility of a polymer in one or a mixture of solvents. In solution, stimuli responsive polymers undergo a conformational, so-called coil-to-globule, transition. Depending on whether the solubility increases below (LCST for lower critical solution temperature [46–49]) or above (UCST for upper critical solution temperature [50–53]) a critical demixing temperature, this class of polymers can be divided into those with LCST or UCST behavior. Physicochemically, these temperature-dependent phase transitions and consequently the term UCST/LCST are exclusively defined for substances, like polymers, in solution. However, the related concepts have been extended to polymers attached to flat or particulate surfaces as well as polymers in star or micelle constitution in the last decades. Hence, thermoresponsive polymers are still intensively investigated regarding to theory, preparation and their potential use [2,43]. However, while polymers with an LCST type phase transition have been extensively studied over the past decades, interest in the equally promising but usually more complex and experimentally much less frequently observed UCST-based phase transitions of polymers has grown strongly in recent years.

The UCST-type thermoresponsive behavior of polymers is governed by oriented polymer–polymer interactions, such as hydrogen bonding or electrostatic interactions. As a function of temperature, these attractive interactions are formed and contribute to phase separation (*T* < UCST) or are increasingly destabilized and allow solvation of the polymer chains (*T* > UCST). Compared to an entropy-driven LCST behavior of polymers, the enthalpy-driven UCST thermoresponse is based on spatially highly directed forces between polymer segments [51–52,54–55]. Understanding the molecular mechanism and thus precisely controlling the complex interplay of attractive interactions enables fine-tuning of the phase transition temperature itself, as well as the sharpness, switching amplitude and reversibility of the transition for a desired application [6,50–51]. Today there are several excellent reviews offering synthetic guidelines for the design of novel thermoresponsive polymers exhibiting UCST type behavior. Seuring et al. [51,54], Niskanen et al. [50], Bansal et al. [52] or Zhao et al. [55] summarize known thermoresponsive building blocks based on their molecular structure and offer interesting design approaches supported by the underlying thermodynamic mixing theory. However, although the influence of certain molecular parameters such as chain length, concentration, pH or low molecular weight additives are discussed, the thermoresponsive behavior of polymers is exclusively considered as free polymer chains in solution. On the other hand, there are several reviews dealing with applications of thermoresponsive polymers. In this context, among others Zarrintaj et al. [56], Mokhtarinia et al. [57] and Sponchioni et al. [58] present a few novel applications of UCST polymers, especially in the biomedical field in comparison to LCST-based approaches [6–7,59–62]. However, these reviews and the summarized research papers mainly focus on the application itself. A systematic investigation of the underlying structure–property relationship of the thermoresponsive polymer has only been conducted to a very small extent.

Despite of first successful applications of UCST polymers, it is still poorly understood how the polymer’s topology is effecting its thermoresponsive behavior until now. So far, there are neither sufficient theoretical models nor extensive experimental work that can comprehensively describe the influence of grafting density, degree of crosslinking or branching as well as the substrate’s influence on the UCST phase transition of grafted polymers. Moreover, for polymers in solution, a generalized thermodynamic description and a standardized characterization of the macroscopic effects via turbidimetry (determination of the cloud point) is available. For thermoresponsive interfaces in the form of polymer brushes on a flat substrate or on nanoparticles, however, there are only very limited comparable analytical characterization methods, which consequently leads to a distortion of terminologies and makes it difficult to compare different studies. Nevertheless, based on selected studies, we want to highlight different topologies of UCST polymers in the subsequent discussion and work out their impact on the thermoresponsive phase transition. In contrast to a large number of previously published studies, the present review does not only focus on the molecular structure of thermoresponsive polymers and their synthesis but also discusses their phase transitions, in terms of structure–property relationships arising from the alignment of the polymer chains in assemblies of constrained mobility. Benefits as well as limitations of selected grafted or crosslinked architectures will be pointed out. However, based on the relatively small number of available studies and sometimes contradictory findings, it is evident that there is a big demand of academic research to develop generalized guidelines for the use of UCST-type polymer topologies especially within multicomponent systems.

The present review will be limited exclusively to stimuli-responsive polymer layers bonded to flat and particulate inorganic surfaces as well as to star polymers and polymer micelles. Only polymer layers that are covalently, i.e., chemically, bonded will be considered in contrast to non-bonded Langmuir–Blodgett or hydrogel films [63–64].

The following contribution is divided into three sections. The review will be preluded with a presentation of the physicochemical aspects of the phase transition of unhindered polymer chains in solution with an LCST or UCST behavior, followed by a consideration of grafted linear polymers, star polymers and micelles having such thermoresponsive characteristics. Because studies about systems with UCST behavior are underrepresented and only sporadically physicochemically analyzed, the focus will be on this type of phase transitions. Additionally, the knowledge about the LCST behavior of the mentioned polymer structures (micelles, star polymers) and grafted configurations (chains anchored on spheric and flat substrates) will be briefly summarized. This review article aims to outline the existing knowledge on thermosensitive polymers from a physicochemical rather than a synthetic point of view in order to gain a deeper understanding of the mechanisms involved in the phase transition with upper critical demixing point versus lower critical demixing point. The fundamental understanding of the polymer–solvent (–surface) interactions and the influence of the polymer morphology is essential for the generation of new polymer-equipped devices with tailored properties for specific applications. At the end of the review article all abbreviations are explained in a separate list.

## Review

### Theory of LCST and UCST

#### Fundamental thermodynamics of binary mixtures

The theoretical considerations about the mixing behavior of a polymer in a solvent, which is often characterized by a limited temperature-dependent miscibility due to the specific properties of macromolecules, is introduced with some fundamental remarks on the thermodynamics during the mixing process [65–66]. From the mathematical description of the law of energy conservation (1st law of thermodynamics) and the assumption that energy cannot be completely converted into work (2nd law of thermodynamics), a criterion for the spontaneity of processes in general, and the mixing processes discussed here, has been thermodynamically formulated. Mathematically, this description results in the Gibbs energy of mixing Δ_m_*G*, which is linked to the enthalpy of mixing *∆*_m_*H* and the entropy of mixing *∆*_m_*S* via the following well-known connection:

[1] $$\Delta_{\text{m}}G=\Delta_{\text{m}}H-\Delta_{\text{m}}S$$

This means that for positive values of Δ_m_*G*, segregation occurs and for negative Δ_m_*G*, mixing occurs. For an ideal binary mixture of components A and B, this equation can be converted into the following form by comparing the Gibbs energy before and after mixing using the chemical potentials µ:

[2] $$\Delta_{\text{m}}G=n\cdot R\cdot T\cdot \left(\varphi_{\text{A}}\cdot \ln\varphi_{\text{A}}+\varphi_{\text{B}}\cdot \ln\varphi_{\text{B}}\right)$$

with *n* = *n*_A_ + *n*_B_. It should be noted that the essential property of an ideal mixture of liquids is not the exclusion of all interactions as it is assumed for the mixing of ideal gases. In an ideal solution, there are interactions between the components A and B, but the average interaction energy between A and B in the mixture is the same as the average energy of interaction between the single components in the pure liquids. Therefore, the enthalpy of mixing, the difference between the enthalpy of the mixture and the enthalpy of the pure components, *∆*_m_*H* is zero and from the comparison of Equation 1 and Equation 2 we obtain for the entropy of mixing:

[3] $$\Delta_{\text{m}}S=-R\cdot T\cdot \left(\varphi_{\text{A}}\cdot \ln\varphi_{\text{A}}+\varphi_{\text{B}}\cdot \ln\varphi_{\text{B}}\right)$$

**Figure 1:**
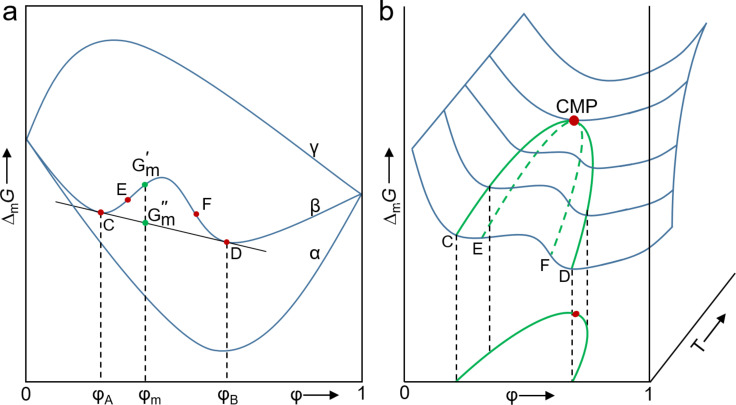
(a) Schematic representation of the phase stability of a binary mixture based on the free enthalpy of mixing *∆*_m_*G* as a function of composition at constant pressure and temperature. Function α shows the curve for an ideal mixture with complete miscibility. Curve β corresponds to a system with a miscibility gap. Here, for example, a mixture of composition φ_m_ decomposes into the corresponding phases A and B with the corresponding volume fractions φ_A_ and φ_B_. The mixture can thereby lower its energy from 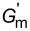 to 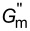. The curve γ shows the behavior with complete immiscibility. (b) Schematic representation of the phase stability of a binary mixture with limited miscibility in a spatial φ–Δ_m_*G*−*T* diagram with projection of the Gibbs energy of mixing curves onto the φ–*T* plane for the construction of the known temperature-composition curves is exemplarily shown for a system with an upper critical demixing point and corresponding upper critical solution temperature (UCST).

Thus, the driving force of the mixing process is the increase in disorder due to the mixing of the particles of both species. *∆*_m_*G* is therefore always negative for ideal mixtures of any composition with a minimum at a certain ratio A–B and is always smaller than the Gibbs energy for a heterogeneous system over the entire concentration range (illustrated in Figure 1a, curve α). The Gibbs energy of mixing cannot be reduced by demixing, so that A and B are miscible with each other in any ratio. The mixture is always more stable than the pure components. In contrast, the curve γ shows an example for the behavior of *∆*_m_*G* of a real mixture. Here, the Gibbs energy of the single-phase state is always higher than the mixture of the two components. Therefore, the two liquids are immiscible with each other over the whole concentration range. On the molecular level, these real mixtures are characterized by interactions A–A, A–B as well as B–B, which are all different from each other. The mixing process is thus accompanied by an enthalpy change and possibly by an additional entropy contribution. The free energy of mixing can thus become positive (spontaneous segregation) if the mixing process is strongly endothermic (large positive value for *∆*_m_*H*) and *∆*_m_*S* is negative (for example, due to reorganization of the molecules, which can lead to a more ordered system).

In addition to systems that are immiscible over the complete composition range, components A and B may also be miscible only in a specific range of possible compositions. This phenomenon is also called partially miscibility. In this case, a mixing gap is formed in which the binary system is characterized by instability (Figure 1, curve β). In this region, *∆*_m_*G* exhibits a maximum and two inflection points (E and F) as a function of composition. This region cannot be realized by “equilibrium” experiments and can only be captured mathematically. These points are also called spinodal points. In addition, two further minima exist. By applying the double tangent to these two points C and D, the composition of the coexisting phases of the hypothetical mixture with the composition *x*_m_ can be obtained. The region between C and D, also called binodal points, and the inflection points is a metastable region. Mathematically, this results in the following instability criterion:

[4] $$\frac{\partial \Delta_{\text{m}}G}{\partial x}=0;\;\frac{\partial^{2}\Delta_{\text{m}}G}{\partial x^{2}}=0$$

These equations are valid for φ_1_ as well as for φ_2_. The point where binodals and spinodals touch, i.e., their values become equal, is called the critical point (Figure 1b). The criterion is that in this point the third derivative of the Gibbs energy after composition is zero:

[5] $$\frac{\partial^{3}\Delta_{\text{m}}G}{\partial x^{3}}=0$$

In many binary systems, the miscibility of two components is temperature dependent. Depending on whether a miscibility gap occurs above or below a certain temperature, a distinction is made between mixtures with an upper critical solution temperature (UCST) or a lower critical solution temperature (LCST). Figure 1b demonstrates the schematic projection of several functions of *∆*_m_*G* at different concentrations and temperatures into a commonly represented *T*−φ diagram using the example of a system with a UCST.

As described above, limited miscibility occurs whenever the mixture exhibits deviations from the ideal state. This means that *∆*_m_*G* is no longer exclusively dependent on the entropy of mixing (Equation 3). In addition, a value for the enthalpy of mixing *∆*_m_*H*, which describes the energetic interactions, must be included in the consideration. Flory and Huggins did significant work on this field, which is widely used in the description of polymer solutions [67–70]. For the enthalpic description, they introduced the interaction parameter χ, which is defined over the intracomponent interactions ε_AA_ and ε_BB_ between the particles of the pure components and intercomponent interactions ε_AB_ between the components among each other. Whether limited miscibility occurs depends on the interplay between these inter- and intracomponent forces. According to Flory and Huggins the χ*-*parameter was derived via a lattice model of statistical thermodynamics and is defined as follows:

[6] $$\chi =-\frac{z}{2kT}\cdot \left(\varepsilon_{\text{AA}}+\varepsilon_{\text{BB}}-2\varepsilon_{\text{AB}}\right)$$

with *k* as the Boltzmann constant, *z* as the (average) number of contacts per molecule and *T* as the temperature. Accordingly, the following relationship is obtained for the enthalpy of mixing:

[7] $$\Delta_{\text{m}}H=R\cdot T\cdot \chi \cdot \varphi_{\text{A}}\cdot \varphi_{\text{B}}$$

By combination of this equation with Equation 3 for the entropy and Equation 1, a mathematical relationship can be derived for *∆*_m_*G*, which is also the mathematical description for the curves α, β, and γ in Figure 1. In the case of an ideal mixture, the χ*-*parameter is zero and curve α is obtained. Because of interactions between the components in nonideal two component systems, the χ*-*parameter takes positive or negative values depending on whether the intracomponent interaction is smaller or larger in comparison to the intercomponent interaction. The miscibility, i.e., whether *∆*_m_*G* becomes negative, is decided by the relationship between *∆*_m_*H* and *∆*_m_*S* and the temperature, which is illustrated in Figure 1.

#### Thermodynamics of polymer–solvent mixtures

Based on these fundamental considerations, these thermodynamic relationships for the mixing process of two liquids are now transferred to free polymer chains in a solvent. For simplicity, a monodisperse polymer is assumed, which, in a first approximation, should behave like a liquid in a solvent. The dissolution of polymers is characterized by strong interactions among the polymer chains and with the solvent molecules. For a polymer to dissolve well in a solvent, the intermolecular forces between the polymer chains on one side and between the solvent molecules on the other side must be smaller than the effective forces between polymer and solvent molecules among each other. If this condition is fulfilled, solvation of the polymer takes place. First, cohesive forces between the solvent molecules and the polymer chains must be overcome. At the same time, polymer chains come into contact with solvent molecules, which leads to the release of solvation energies or adhesion energies. If the Gibbs energy of the individual components (polymer and solvent) is higher than that of the homogeneous mixture, *∆*_m_*G* is less than zero. The components mix into each other. Conversely, if *∆*_m_*G* > 0, complete dissolution of the polymer cannot occur. Coexisting, separate phases with a high and a low polymer content are formed. Experimental and theoretical studies show that there is both a concentration and temperature dependence of χ [71–74]. Consequently, for the exact description of complex phenomena of the dissolution of polymers, an extension of the basic theory is necessary, which results in a various number of tailored models [75–77], like the Flory–Huggins–van Santen (FHS) model. The FHS model shows that the combinatorial entropy of mixing is much smaller for macromolecules than for compounds with a low molar mass. For modelling of phase diagrams with LCST and/or UCST, it is common practice to replace χ by a semiempirical interaction parameter *g*, which is both temperature and concentration dependent [78–79] and can be modelled by using the following polynomial in φ_2_ (with *k* as index of summation).

[8] $$g=\sum_{k=o}^{n}g_{k}\cdot \varphi_{2}^{k}\;k=0,1,2,3,...,n$$

[9] $$g_{k}=g_{k0}+\frac{g_{k1}}{T}+g_{k2}\cdot k\;g_{k0},g_{k1},g_{k2}=const.$$

**Figure 2:**
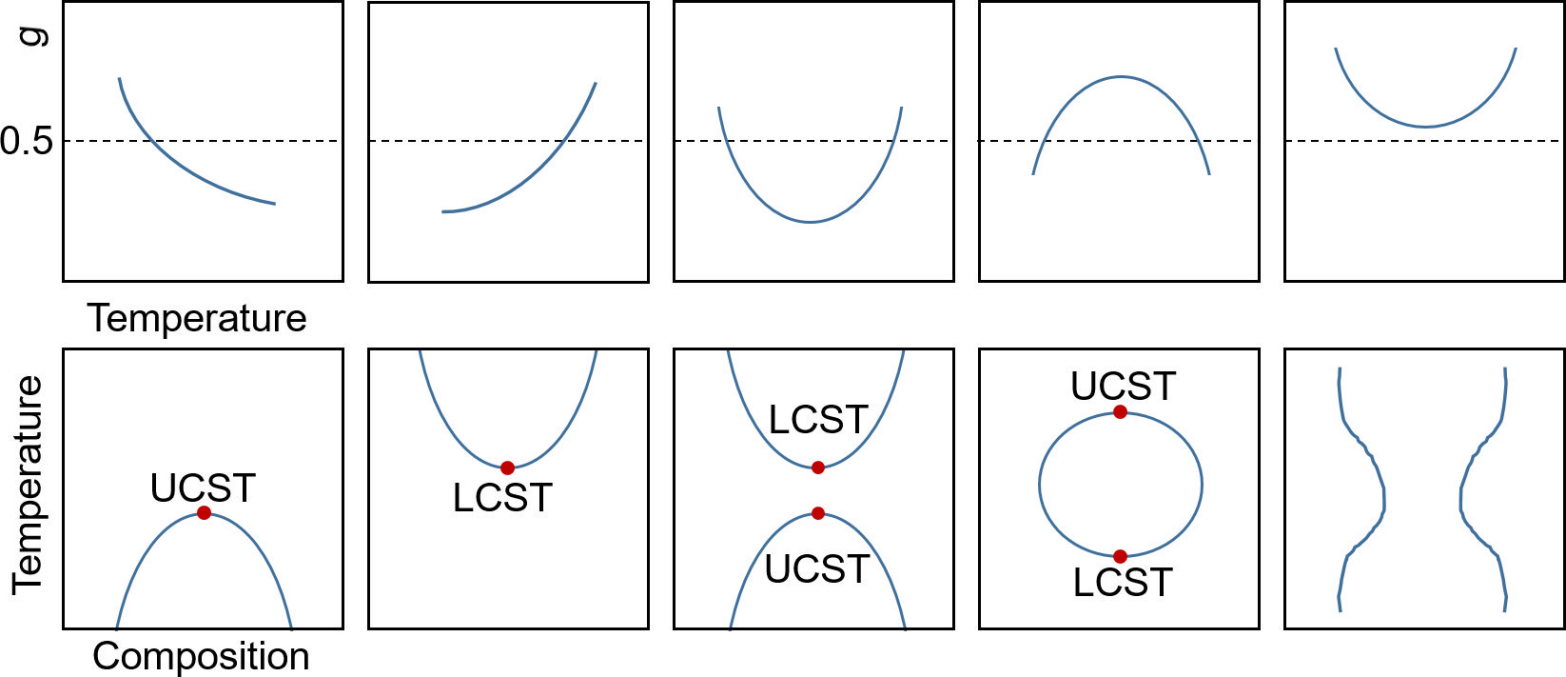
Illustration of the relationship between the type of miscibility gap and the temperature dependence on the *g*-parameter. In the temperature–composition diagrams the binodal lines are shown. Figure 2 redrawn from [51].

Due to a complex temperature dependency of *g* (denoted as *gk* in Equation 9) the polymer can show an upper or lower critical demixing point or a combination of both (Figure 2). In the discussion so far, it was assumed that the investigated polymer solution is strictly binary and consists only of a solvent and the polymer with one molar mass. However, this is not true for any real polymer solution. Every polymer always exhibits a molar mass distribution. Therefore, a quasi-binary mixture is usually considered. It is possible to measure the cloud-points, but the obtained cloud-point (*T*_cp_) curve differs from a simple bimodal. Thus, the critical point (LCST or UCST) of one specific polymer with distinct *P*_n_ is not the extremum and is found at higher polymer concentrations in practice. The maximum of the cloud-point curve shifted to higher temperatures and to the solvent-rich region. The theoretical model was accordingly extended to account for polydispersity [80–82].

In general, the determination of cloud-points with respect to a thermally induced phase transition is a widely discussed phenomenon in the literature. At this point we would like to refer to an excellent work by Hoogenboom and co-workers [46]. We will only briefly discuss the definition of *T*_cp_ and how it differs from LCST and UCST. The *T*_cp_ is defined as the temperature at which the phase transition of a polymer solution at a certain concentration from the soluble state to the collapsed state occurs, accompanied by the turbidity of the solution. This temperature can be determined by various methods, such as turbidimetry [83], ^1^H NMR spectrosocopy [84], and dynamic light scattering [85–86]. In this context, the cloud-point is the phase transition temperature at a certain polymer concentration, which can be located at any point on the binodal curve, and therefore, the polymer concentration must be specified sets in the cloud-point determination. It should be noted that *T*_cp_ is not equivalent with LCST or UCST, since LCST corresponds to the minimum temperature value or UCST corresponds to the maximum temperature value of the binodal curve. In other words, the LCST is the lowest value of *T*_cp_ in the phase diagram and the UCST is the highest value of *T*_cp_. The cloud-point curve does not exactly match the binodal curve in the overall phase diagram [87]. This difference between cloud-points and the binodal curve is related to kinetic aspects of determining *T*_cp_ versus the thermodynamic binodal curve, as well as to the limitations of the turbidity measurement, since it only detects polymer agglomerates that are sufficiently dehydrated and large enough. In addition, the cloud-point depends on the method used for the determination. In addition to these differences between LCST/UCST and *T*_cp_ for polymers in solution, the differences to polymers grafted on surfaces must be considered. These include, in particular, the strong interaction between the grafted chains caused by their close distances to each other and the limitation in their degrees of freedom of movement due to the substrate geometry. In addition, each grafted film has a density- and thus a concentration profile, which makes it difficult to define a quantity corresponding to the *T*_cp_. The exact concentration in a grafted polymer film is not known and can only be indirectly given by the grafting density. Thus, the phase transition temperature observed by methods such as in situ ellipsometry is by definition different from such temperatures determined in solution.

**Figure 3:**
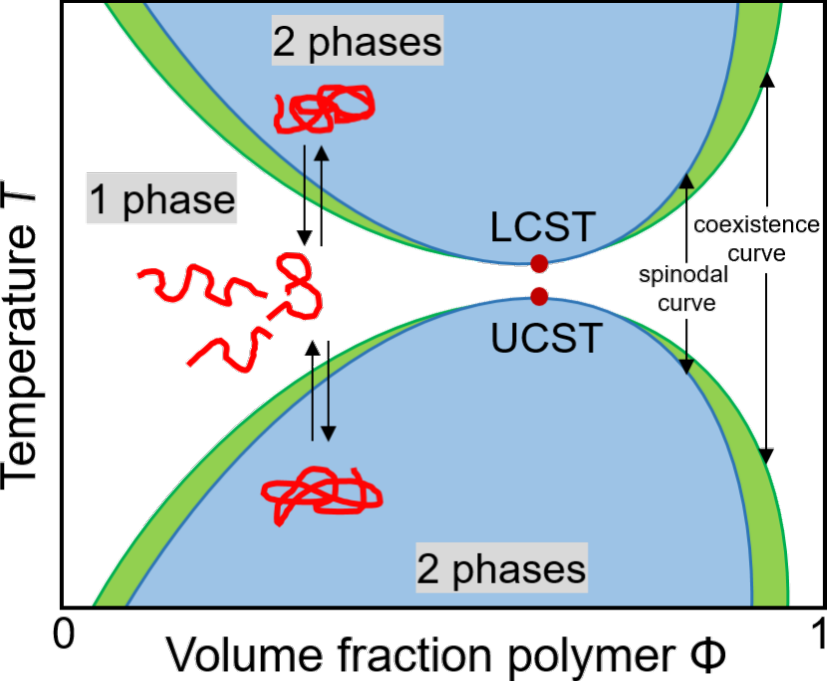
Schematically pictured phase diagram of a binary mixture composed of a dissolved polymer with a LCST and an UCST miscibility gap, respectively.

The critical points in the isobaric phase diagram, where temperature is plotted versus composition, are denoted as lower critical solution temperature or upper critical solution temperature. In the case of a polymer with a UCST, the polymer is in a phase-separated, collapsed state below this temperature. Heating increases the solubility until only one phase is present above the UCST. Schematically, the chains are in stretched conformation and soluble in all proportions. In contrast, the LCST is the temperature above which the polymer is insoluble (Figure 3).

The reason of this described UCST behavior lies mainly in enthalpic effects [50–51]. From a thermodynamic point of view, *∆*_m_*H* and *∆*_m_*S* of the mixing process are positive for UCST polymers. If the temperature curve is considered, it can be seen that at a low temperature *∆*_m_*H* is larger than the *T∙∆_m_S* term. According to Equation 1, *∆*_m_*G* is positive and the polymer is insoluble. When the temperature is higher than the temperature of the associated binodal at a given composition, the expression *T·∆*_m_*S* exeeds *∆*_m_*H* and *∆*_m_*G* becomes negative and the polymer dissolves. This phenomenon occurs for polymers whose interactions with the solvent or between the chains are characterized by strong dipol–dipol interactions, such as Coulomb forces, or by hydrogen bonds. Hence, on the one hand, strongly charged zwitterionic polymers, such as PDMAPS and its structural analogues [88–91], and on the other hand, polymers with strong hydrogen acceptor and donor units, like the uncharged PNAGA [92–96], show a pronounced UCST.

In contrast, the solubilization process of a polymer with LCST behavior is an entropic effect characterized by negative *∆*_m_*H* and *∆*_m_*S* values. The essential structural feature of such polymers is their amphiphilic structure of hydrophobic domains and hydrophilic groups, which can form hydrogen bonds with water. These interactions result in a highly ordered hydration shell when the macromolecule is in solution. The best known example of a phase transition with an LCST is PNIPAAm with its hydrophilic isopropyl groups interacting with water molecules [48,97–99]. From a thermodynamic point of view, the hydrogen bonds between the polymer and the water molecules lead to a negative value for *∆*_m_*H* and, due to the ordered hydration structures, to a negative entropy of mixing. As soon as the turbidity temperature is reached, the hydrophobic effect becomes dominant and water molecules are released, leading to a collapse of the hydration shell and a significant increase in entropy. The intra- and intermolecular interactions between polymer segments are favored and the polymer begins to precipitate. A comparison of the calorimetrically determined enthalpies of mixture *∆*_m_*H* for PNAGA and PNIPAAm reveals the different mechanisms. The entropically driven LCST phase transition of PNIPAAm has a substantially smaller value for *∆*_m_*H* (≈5 kJ/mol) [100–101] than the enthalpically based UCST transition of PNAGA (≈90 kJ/mol) [92], which additionally indicates stronger polymer–polymer interactions for UCST polymers.

#### Phase transition behavior of particular polymer architectures

The relationships described above concerning the segregation behavior of a polymer below or above a certain temperature and the associated formation of a miscibility gap were initially considered only for free polymer chains in solution. In the following section particular polymer arrangements and architectures will be briefly introduced concerning the relationship between their morphology and the temperature-induced phase transition. These polymer arrangements of constrained mobility include star polymers, micelles and grafted linear polymers on flat surfaces as well as on nanoparticles. In general, these four polymer arrangements are characterized by the fact that the mobility of the polymer chains is constrained, in contrast to free polymer chains in solution. Due to the substrate geometry in the case of grafted polymers or due to their arrangement through their structure in the case of micelles and star polymers, the polymers are forced into a certain structure and their degrees of freedom of movement are thus limited. As a result, extremely high segment densities are sometimes generated. In the case of grafted polymers, this depends on the grafting density and substrate geometry. In micelles, the local concentration depends on the total chain length, as in star polymers, and on the ratio of hydrophilic and hydrophobic components. A phase diagram, as described above, describes the mixing behavior over the entire concentration range, i.e., from 0 to 100%, which, however, is never completely reached in the four polymer arrangements studied. Therefore, we cannot speak here of phase diagrams in the classical sense, as well as about a UCST and LCST in the strict sense. Nevertheless, the micelles, star polymers and grafted polymers show a responsive behavior, which will now be discussed in general. The main focus is on fundamental trends. Subsequently, these trends are applied to numerous UCST systems.

**Figure 4:**
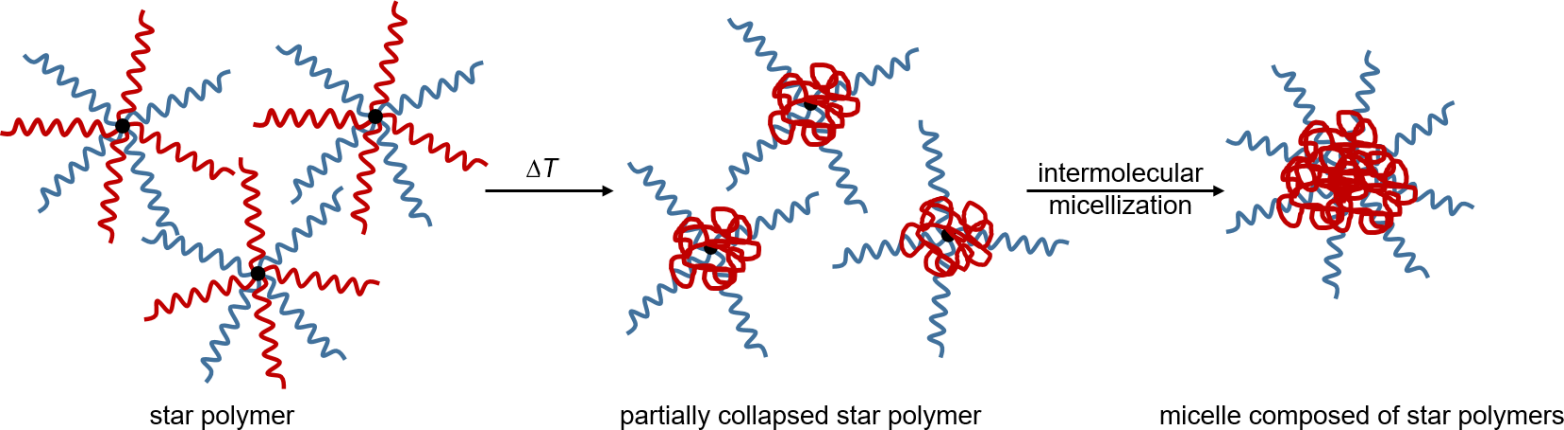
Schematic illustration of a thermo-induced swelling behavior of a star polymer composed of responsive (red) and nonresponsive (blue) polymer chains and their subsequent intermolecular micellization. Figure 4 redrawn from [108].

**Definition and phase transition behavior of star polymers and polymeric micelles:** So far, only the phase transition for free polymer chains in solution has been studied. The arrangement of polymer chains can form superstructures that have a direct influence on the physical properties, such as mobility and intermolecular interactions, and thus also on the position and width of the phase transition. A topologically simple way to restrict the mobility of the polymer chains is the radial, covalent fixation of at least three polymer chains around a core. If the dimension of the core is much smaller than the length of the arms, that is, the root-mean square end-to-end distance, this arrangement is called star polymer [102–107]. These "arms" extend into the surrounding solvent and can interact with it. If thermoresponsive polymers are used for this purpose, the swelling behavior can be thermo-induced. In combination with other nonresponsive polymers the corona of the star polymer can be modulated resulting in an intermolecular micellization [108], which is illustrated in Figure 4. In contrast to free polymer chains in solution, branched polymers have an enhanced segmental density with the same molar mass and are therefore more similar to the hard sphere model [109]. Due to the confined structure, star polymers possess distinct physicochemical properties such as a low viscosity, high density of polymer segments and functional groups as well as a smaller hydrodynamic radius and larger diffusion coefficient compared to linear polymer chains in solution.

**Figure 5:**
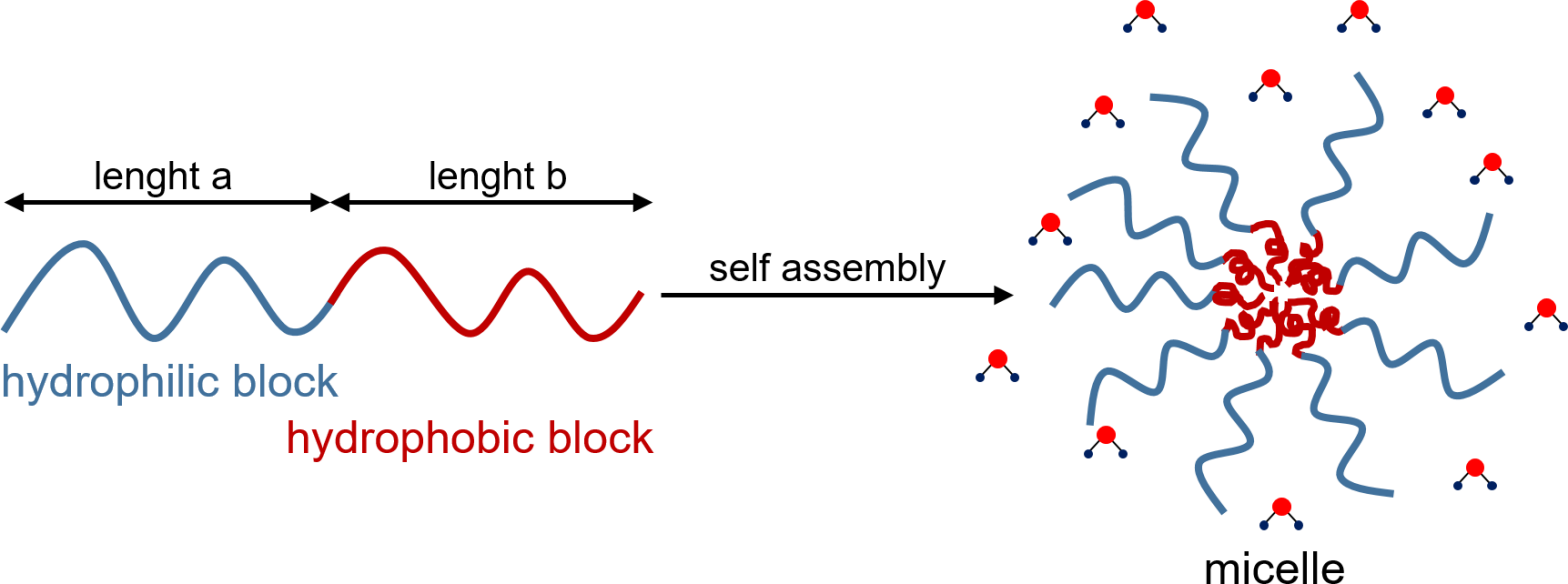
Schematic illustration of self-assembly of block copolymer amphiphiles in a polar medium.

Amphiphilic AB-type copolymers spontaneously form micelle structures above a critical concentration by self-assembly due to their structure consisting of hydrophilic and hydrophobic units [110–113]. In the case of rather large corona blocks compared to the core-forming blocks, such structures are usually star-shaped and spherically composed of many individual chains by noncovalent interchain interactions in contrast to star-shaped polymers. Thus, in a polar solvent, such as water, the hydrophobic block forms an anhydrous core. The hydrophilic chains stretch into the solvent in the shape of a swollen corona (Figure 5). The size, shape and dynamics of the micelle can be essentially tuned by the absolute length of the block copolymer and the relative length of the blocks to each other and the glass transition temperature. If a block is composed of a thermoresponsive polymer, the amphiphilic character and self-assembly ability can be altered by changing the temperature. The thermoresponsive block collapses or swells depending on the temperature resulting in a reversibly switching behavior of structure and shape of the micelle.

**Figure 6:**
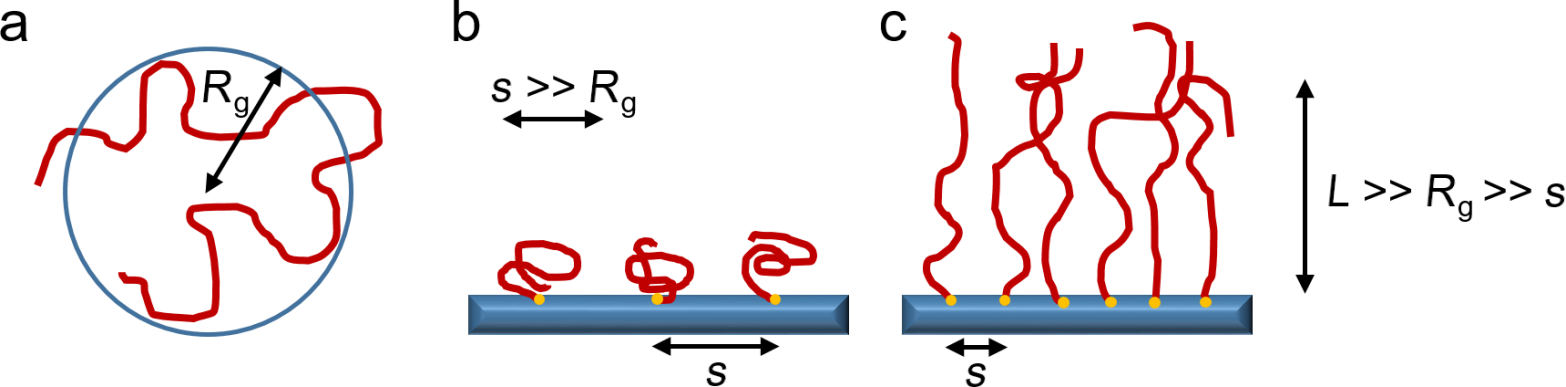
Schematic comparison of the size and conformation between free polymer chains (a), grafted polymer chains in mushroom regime (b) and polymer chains grafted on a surface in the brush conformation (*R*_g_ – radius of gyration, *s* – distance between grafting points, *L* – brush height). Figure 6 redrawn from [117].

**Definition and phase transition behavior of polymers grafted on flat and particular surfaces:** For numerous applications, immobilization of the polymer chains is desirable. In solution, a phase diagram is uniquely defined and can be easily determined by turbidity measurements, for instance. On a surface, however, conventional segregation is not possible due to the hindered mobility of the polymer chains. In contrast, external triggers, such as temperature, can more or less switch between a stretched and a collapsed brush conformation, leading to a change in macroscopic properties, such as contact angle or layer thickness, in contrast to the cloud point for free polymer chains. The influence of covalent bonding on a flat or curved surface will be considered using the example of the polymer brush structure, which has been intensively studied both experimentally and theoretically [114–117]. In general, these influencing parameters include the curvature and general properties of the substrate, the grafting density and chain end effects. A polymer brush is defined as a dense array of flexible polymer chains chemically or physically attached to a surface through one end of the chain [118]. The distance between the attachment points is decisive for the properties of the polymer film and its differences from free polymer chains (Figure 6). If the average distance between the anchor points is smaller than the undisturbed gyration radius *R_g_* of the free chains, the polymer chains stretch to the so-called polymer brush conformation, due to the repulsive segment–segment interaction, in contrast to the so called “mushroom regime”. That means, the intermolecular interactions in the brush dominate over the intramolecular ones. As a result, the chain conformation within the brush structure is always stretched compared to unattached pendants.

**Figure 7:**
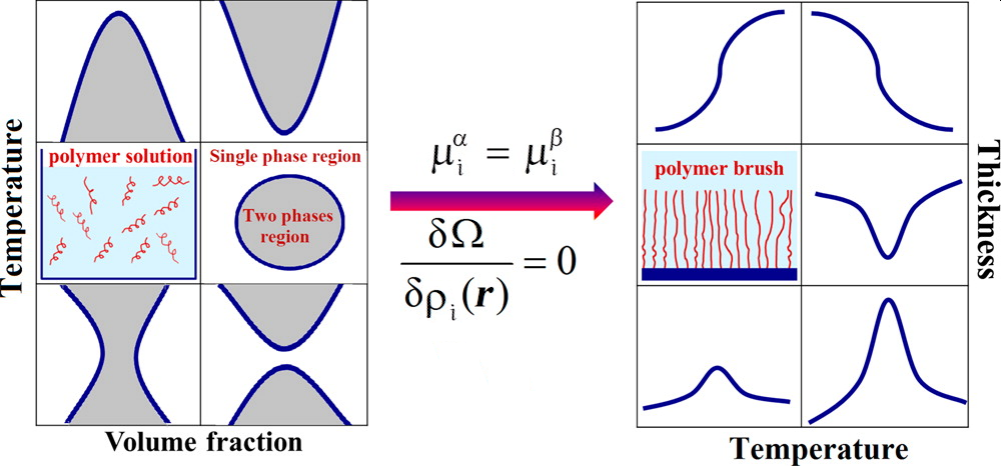
Comparison of the possible phase diagrams of a polymer in solution with partially miscibility and the corresponding temperature–thickness curve, showing the swelling behavior of the polymer brushes as a function of the temperature change (μ – chemical potential, *i* – component, Ω – potential of the grand canonical ensemble, *ρ* – density distribution, *r* – chain length). These graphs were simulated using lattice density functional theory. Figure 7 was reprinted with permission from [124]. Copyright 2014 American Chemical Society. This content is not subject to CC BY 4.0.

The first theoretical investigations into the swelling behavior of polymer brushes based on the work of de Gennes [119–120] and Alexander [121]. In this theoretical work scaling laws were derived for uniformly stretched neutral polymer chains in nonpolar solvents that form a brush with fairly uniform density (“box-model”). The scaling laws allow the description of the swollen layer thicknesses as a function of the grafting density and the molecular weight of the polymers. Using the self-consistent field theory, it was possible to describe the nonequally and nonuniformly stretching behavior in order to minimize the whole conformational entropy loss [122–123]. Using further theoretic models and methods, such as the lattice density functional theory, the temperature-dependent swelling behavior of a polymer brush could be simulated in comparison to the corresponding phase diagrams of the free, unbound polymer chains in solution [124]. The corresponding diagrams are summarized in Figure 7 and show the typical thickness–temperature graphs for polymer brushes.

With regard to the interactions between the chains, a distinction is made between so-called classical polymer brushes, in which only van der Waals interactions dominate between the chains, nonclassical neutral brushes, in which additional specific interactions occur between the chains themselves and with the solvent including structuring effects in hydrogen bonding solvents, and polyelectrolyte brushes, whose behavior is additionally determined by long-range Coulomb interactions [114,116]. The main difference between classical and nonclassical polymer brushes is the role of the solvent, which has an influence on the internal structure of the polymer film. This leads to different distributions of the polymer segments and therefore to different polymer profiles in vertical extension to the surface. This effect has a direct impact on the occurrence and width of phase separation with an LCST or UCST and its dependence on brush parameters, such as local concentration, and thus on its influenceability. It has often been shown experimentally that the most pronounced conformational response of polymer brushes is achieved at moderate grafting densities [125]. Furthermore, the collapse of end-tethered assemblies is generally weaker and broader due to the interaction between the chains compared to isolated chains in solution [123,126]. In the case of classical polymer brushes, i.e., nonpolar systems such as polystyrene brushes, the transition from a swollen state to a collapsed state is characterized by the change of the segment distribution from a parabolic to a step profile and a rearrangement of the chain ends [126]. This collapse can be described by the classical Flory–Huggins model. It follows that this vertical collapse above the LCST or below the UCST is merely a shrinkage and not a discontinuous transition [70,114,125,127]. Unlike free, unbound polymer chains, where the degree of dilution can achieve any composition in the phase diagram, the extent of dilution in brushes is limited and a function of grafting density and temperature. For phase segregation, the concentration of segments in a brush must be in the semi-dilute region. The associated concentration is generally much higher than required for the critical temperature. Hence, the contraction of the brush never crosses the coexistence curve with decreasing temperature [114]. Due to the special arrangement of the chains on a surface and the intermolecular interaction that occurs, vertical phase separation is suppressed in systems such as polystyrene in cyclohexane, in contrast to free dissolved chains [128]. However, at vanishingly small concentrations, classical brushes always exhibit a behavior resembling phase separation with UCST. Unlike this classical polymer brush, the χ-parameter for water-soluble polymers depends on both temperature and concentration as well as on φ [71–74,129–130]. This dependency results in a shift of the critical point and a change of the shape of the coexistence curve enveloping the two-phase region. Because of the specific interactions between water and polymer chains, water-soluble polymers can have a critical point at any concentration, which often corresponds to a miscibility gap with a LCST [74], in contrast to nonpolar brushes in an organic solvent (like polystyrene in cyclohexane), which always show an UCST. This critical point is usually located in the semi-dilute regime and the brush can contract discontinuously or continuously [114,131]. The more complex dependencies on χ leads to a bilayer-type profile, as has been shown for PNIPAAm [132–133], since the near-surface segments tend to segregate compared to the outer region. This results in a broadening of the temperature range in which the polymer chains collapse. The third class of brushes are films composed of polyelectrolytes in which long-range Coulomb interactions are dominant [134–135]. Here the charges of the polymer chains and therefore the intermolecular repulsions are compensated by counterions. Due to this counterion condensation, the majority of ions are located inside the brush. Therefore, the interface is in a charged state, viewed from the outside, which can be easily observed by, e.g., zeta potential measurements. Two scaling regimes can be defined as limiting cases using the specific localization of the counterions that results from additionally added salt: the salted brush and osmotic brush [136–138]. These additional counterions, in addition to pH changes, have significant influence on the swelling behavior in the case of weak polyelectrolytes. In a salted brush, the concentration outside and inside the brush is approximately the same and the swelling properties are determined by excluded volume interactions between the segments. In an osmotic brush, the concentration of trapped counterions inside the brush is greater than the concentration outside. The resulting osmotic pressure depends on the interconnected polymer segments, the chain elasticity and the trapped counterions.

The previous remarks on the swelling behavior of polymer brushes on planar surfaces can essentially be transferred to curved surfaces, that is, to coated particles. Particulate systems [115,117] are characterized by the fact that a certain number of coated surfaces interact with each other. The temperature-dependent swelling influences the interactions between the functionalized particles. In addition to the described factors influencing the position and width of the critical point, the curvature must be considered for particulate systems. A general trend is that the cloud points of responsive nanoparticles are smaller than compared to free polymer chains [139]. The size of the nanoparticle core directly affects the thermal response. Thus, it was found that with increasing particle size, the LCST decreases [140]. The reason for this is the strong dependence of the free space per polymer chain at the same grafting density on the degree of curvature [115].

### Responsive polymers with LCST behavior

The LCST behavior of the mentioned polymer arrangements is a very intensively studied phenomenon in the literature. There are many different polymers that exhibit a phase transition with LCST, such as PNIPAAm, PEGMA, POX, PDMAEMA, PDEAEMA, PDEAEAM, PMEMA and PDEAAM. The review of Roy et al. presents a comprehensive list of investigated polymers with a LCST-like behavior [2]. The influence of structure and properties of the polymers as well as their arrangement on the location and width of the phase transition has also been studied in numerous examples. Therefore, at this point we will only refer to published reviews on this topic. Table 1 summarizes the work on micellar structures, star polymers and polymers grafted on particles or on planar surfaces as well as their discussed application without the claim of completeness. Due to the large number of investigated polymers with LCST behavior, only a few examples are summarized in Table 1 to illustrate the focus of research, which lies on synthesis, fundamental understanding of the relationship between polymer arrangement and architecture and the phase transition behavior as well as on real application and their potential in different fields of biomedicine, sensing or catalysis, for instance. In addition, some review articles on the respective architectures and polymers in principle are listed.

**Table 1:** Overview of the most significant polymers with LCST behavior ordered by the polymer arrangements star polymer, polymer micelles as well as polymers covalently grafted on nanoparticles and flat surfaces.

Polymer arrangements	Polymer	Object of research/highlighted application	reference

star polymers [102–103,106]	*PNIPAAm-based polymers* [102]		
	PNIPAAm-PDEAEMA	synthesis/analysis	[141–142]
	PNIPAAm-PS	synthesis/analysis	[143]
	PNIPAAm-PEG	synthesis/analysis	[108]
	PEG-PDEAEMA-PNIPAAm	synthesis/analysis	[144]
	PEG-PNIPAAm-Plys/PAA	synthesis/analysis	[145]
	PS-PCL-PNIPAA	synthesis/analysis	[146]
	PEG-PtBMA-PNIPAAm	synthesis/analysis	[147]
	PS-PNIPAAm-PDMAEMA	synthesis/analysis	[148]
	PNIPAAm-P4VP	synthesis/analysis	[149]
	PS-PNIPAAm-P4VP	synthesis/analysis	[102,149]
	PAA-PNIPAAm	nanocarrier/molecule delivery; synthesis	[105]
	PCL-PNIPAAm	nanocarrier/drug delivery, synthesis	[150]
	PTEGDA-PNIPAAm-PNMA	drug delivery, synthesis	[151]
	PNASME-PNIPAAm	synthesis/analysis	[152]
	*POX-based star polymers*		
	PBOX-PEtOX	synthesis/analysis	[153]
	PEtOX- PIPOX	synthesis/analysis	[154]
	PIPOX	synthesis/analysis	[155–156]
	*PEG or PEG derivates-based star polymers*		
	PEG-PDMAEMA	synthesis/analysis	[157]
	PCL-POEOMA-PMEO_2_M	synthesis/analysis	[158]
	PDEGA-PHEA	synthesis/analysis	[159]
	P(DEGMA-OEGMA-GMA)	as nanolayers for controlled cell sheet detachment, synthesis	[160]
	PDMAEMA-PDEGA	synthesis/analysis	[161]
	*other polymers*		
	MPEP-PCL-PPE	synthesis/analysis	[162]
	PVAc-PNVCL-PNVP	synthesis/analysis	[163]

polymer micelles [4,59,164–165]	*PNIPAAm-based micelles*		
	P4VP-PNIPAAm	catalysis, synthesis	[166]
	PNIPAAm-PDMAAm	drug delivery, synthesis	[167]
	PNIPAAm-DNA	synthesis/analysis	[168]
	PNIPAAm-PHPMA-PEG	synthesis/analysis	[169]
	PNIPAAm-PBMA	drug delivery, synthesis	[170]
	PNIPAAm-PS	synthesis/analysis	[171–172]
	PNIPAAm-HPG		[173]
	*POX-based micelles* [174–175]		
	PEtOX-PPropOX	synthesis/analysis	[176]
	PIPOX-PAMPT	radionuclide delivery, synthesis	[177]
	PMeOX-PIPOX-PBuO	synthesis/analysis	[178]
	PBOX-PEtOX	drug delivery, synthesis	[153]
	PMeOX-PBuOX	synthesis/analysis	[179]
	*other polymers*		
	PDMAEMA-PCL	drug delivery, synthesis	[180]
	PEGMA-PMMA-PDEAEMA	synthesis/analysis	[181]
	PHPMA-PDEGMA	drug delivery, synthesis	[182]

polymers grafted on nanoparticles [4,115,117,164,183–185]	*PNIPAAm-based grafted nanoparticles*		
	PNIPAAm-	preparation/analysis	[186]
	PDMAAm@Fe_3_O_4_	preparation/analysis	[187]
	PNIPAAm-PNHMA@ Fe_3_O_4_	drug release, preparation	[188–191]
	PNIPAAm@SiO_2_	molecule delivery, preparation	[191–194]
	PNIPAAm@Fe_3_O_4_	microfluidic separation and assay; sensing; drug release, preparation	[195–201]
	PNIPAAm@Au	fundamental investigation, preparation, cell up-take control	[200,202]
	PNIPAAm-PAm@Au PNIPAAm-PNVP@Fe_3_O_4_	cell up-take control drug delivery/cell separation, preparation	[203]
	PNIPAAm-PDMAEMA@Fe_3_O_4_/Au	catalysis, preparation	[204]
	PNIPAAm-PAA@UCNP	sensing, preparation	[205]
	PNIPAAm-PMAA-PVP@Fe_2_O_3_	drug delivery, preparation	[206]
	*POX-based grafted nanoparticles* [207]		
	PIPOX@Fe_3_O_4_	preparation/analysis	[208]
	PIPOX-PEtOX@SPION	cell up-take control	[209]
	PEtOX/PPropOX-PVIm/P4VP@Ag	preparation/analysis	[210]
	*PEG or PEG derivates-based grafted nanoparticles* [211]		
	PEG-PDMAEMA@Au	preparation/analysis	[212]
	PEGMA@Au	preparation/analysis	[151,213]
	PMEO_2_MA-OEGMA@Au	protein adsorption, preparation	[214]
	PEGMA-PEG@SiO_2_	catalysis, preparation	[215]
	PEG-PPO@SPIO	protein adsorption, preparation sensing, contrast agent	[216]
	PEG-PPO@ SiO_2_	drug delivery	[217]
	*other polymers*		
	P(HPEI-IBAm)@Au	preparation/analysis	[218–219]
	PVME@Au	preparation/analysis	[220]
	PMDM@Au, glass or SiO_2_	catalysis, sensing, preparation	[221–223]
	PTEGMMA-PMAPMA@SiO_2_	protein adsorption, preparation	[224]

polymers grafted on flat surfaces [3,43,225–226]	*PNIPAAm-based grafted surface* [43,227]		
	PNIPAAm@different flat substrates, such as Au, Si-wafers, glass	preparation, theory, fundamental investigation of swelling behavior, cell/protein adhesion, sensing, environmental application	[132,228–255]
	PNIPAAm-PAA@SiO_2_	protein adhesion, preparation	[256–259]
	PMMA-PBIEM-PNIPAAm	cell up-take control, preparation	[260]
	PNIPAAm-PLAMA@glass	preparation	[261]
	PNIPAAm-PDMAEMA@Al	preparatio	[262]
	PNIPAAm-PNTBA@glass	cell up-take control, preparation	[263]
	PNIPAAm-ODS@glass	microfluidic, preparation	[264]
	PNIPAAm-PEG@Au	cell up-take control, preparation	[265]
	*POX-based grafted surface*		
	PCPOX-PMeOX@SiO_2_	preparation	[228]
	PPropOX@glass	cell up-take control, preparation	[266]
	*PEG or PEG derivates-based grafted surfaces* [43,211]		
	PMEO_2_MA-PDEAEMA@SiO_2_	preparation	[267]
	PMEO_2_MA-POEGMA@SiO_2_	protein adsorption, preparation	[268]
	*other polymers*		
	PMDM@glass	protein adsorption, preparation	[221]
	PVCL-PDMAEMA-PMPC@PDMS	controlled wettability self-cleaning, anti-microbial coating, preparation	[269]

### Responsive polymers with UCST behavior

**Figure 8:**
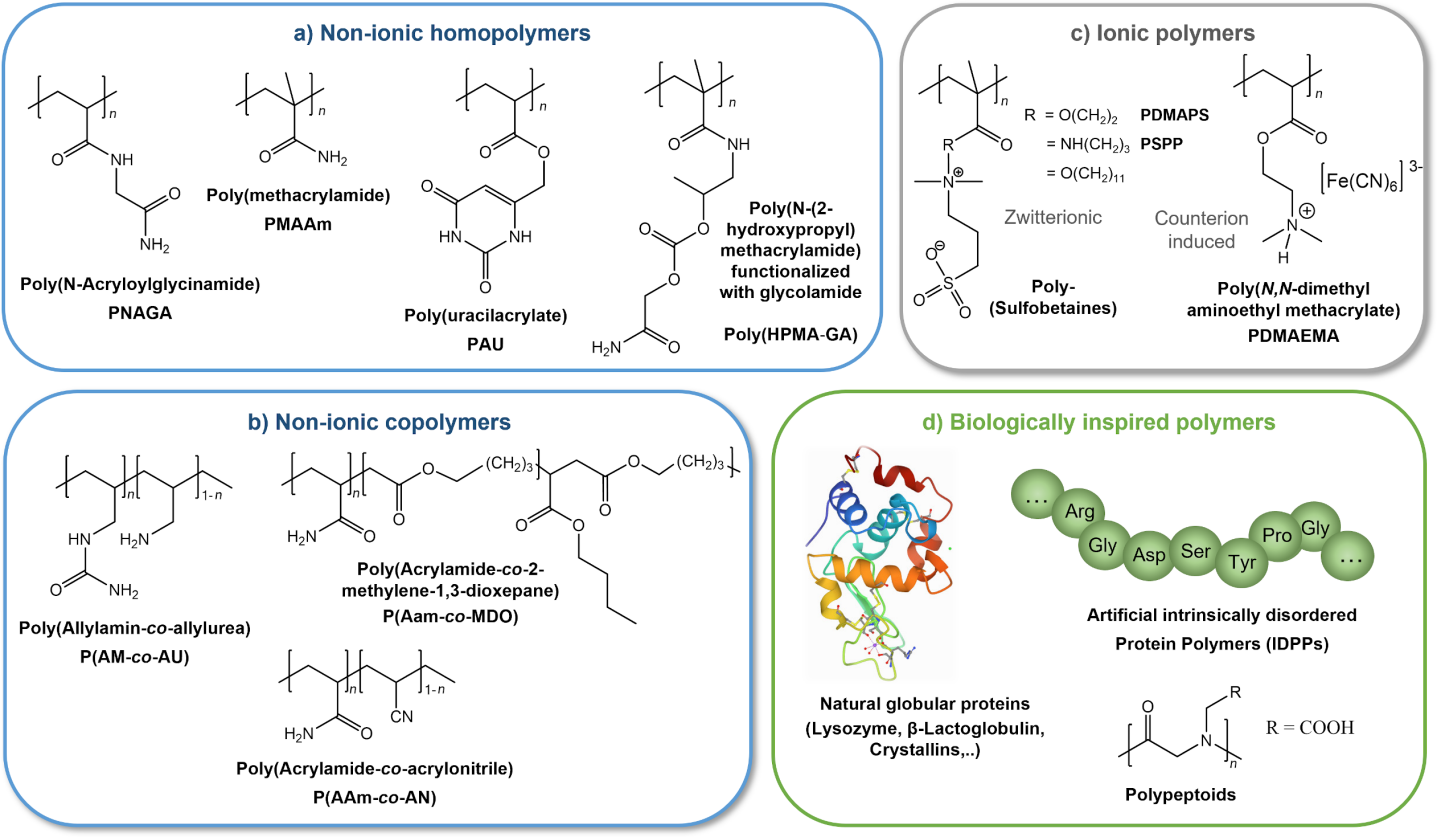
Selection of polymers exhibiting UCST behavior due to hydrogen bonding (blue) divided into homo- (a) and copolymers (b), as well as upon attractive electrostatic interactions (gray, c)). Furthermore, biologically inspired polymer structures with UCST behavior are shown (d), in which thermoresponsiveness arises from a variety of intermolecular interactions. The illustration is intended to highlight several polymer classes with confirmed UCST behavior, without making a claim to completeness. The crystal structure of hen egg white lysozyme in part d) is reprinted from the RCSB Protein Data Bank [270].

In this section, we would like to give the reader a brief overview and update of known polymeric building blocks exhibiting UCST behavior, highlighting in particular recently developed structures (Figure 8). Subsequently, their use in different topologies and assemblies such as star polymers, micelles as well as covalently grafted polymers on flat substrates and nanoparticles will be explained in more detail.

According to the type of supramolecular interactions governing the thermoresponsive behavior UCST type polymers can be classified into two categories. Although various molecular forces such as van der Waals interactions and hydrophobic effects contribute to the thermoresponsiveness of polymers, UCST type behavior is generally governed either by strong hydrogen bonding or by attractive electrostatic interactions [51–52,55].

**UCST resulting from strong hydrogen bonding interactions.** Increasing interest is focussed on nonionic polymers in water, whose UCST behavior is based on hydrogen bonding. Due to their tolerance towards added salt ions, they represent attractive candidates for biomedical applications [50–52]. Among the few known homopolymers of this type, PNAGA, whose UCST behavior was first characterized by Seuring and Agarwal in 2010, is the most widely studied [92–96]. In addition to UCST-type polymers such as poly(methacrylamide) and polyuracilacrylates, which have been known for some time [51–52], Zhang et al. recently presented the synthesis of a novel homopolymer with transiently thermoresponsive behavior [271]. Within a physiologically relevant window, the developed GA-polyHMPA initially exhibits UCST responsiveness, but can subsequently be slowly biodegraded to a fully water-soluble polymer (polyHMPA) via hydrolysis. Initial in vivo studies of a sustained release of either a hydrophilic model protein or a hydrophobic dye entrapped within the collapsed UCST polymer are promising and open new perspectives for the development of the next generation of smart, degradable biomaterials. In addition to the homopolymers mentioned above, more and more copolymers have been discovered, in which UCST behavior can be induced via hydrogen bonding. Interestingly, the individual monomers usually do not exhibit responsive properties. However, the combination within a copolymer leads to a pronounced thermoresponse. A prominent representative of such behavior is poly(acrylamide-*co*-acrylonitrile), which is assembled from a fully soluble, hydrophilic monomer (AAm) and the hydrophobic AN block [51–52,94]. Since the phase transition temperature of the copolymer can be adjusted over a wide temperature range via the ratio of the two monomers, it is frequently used for the development of novel drug release systems [272–276]. Due to the continuous development and improvement of polymerization techniques, more and more diverse copolymers can be synthesized. Very recently, Kertsomboon et al. succeeded in preparing a copolymer (poly(AAm-*co*-MDO)) of acrylamide and a degradable, hydrophobic monomer based on a ring opening polymerisation of cyclic ketene acetals [277]. This copolymer also showed a well-controllable reversible UCST behavior in aqueous solutions. In 2020, Zhao et al. reported that by incorporating azobenzene functionalities into polyacrylamide copolymers, the responsive UCST behavior was furthermore tuneable via light irradiation as well as by host molecule (α-cyclodextrin) complexation [278]. Due to the high number of proton acceptor/donor sites, ureido-modified copolymers such as poly(allylamine-*co*-allylurea) are also becoming more and more important. The critical phase transition temperature can often be finely tuned under physiologically relevant pH and salt conditions via the hydrophilicity of the co-monomers and their molar ratio [52,55,279].

**UCST resulting from attractive electrostatic interactions.** The thermoresponsive behavior of zwitterionic polymers, in contrast to the previously mentioned UCST type polymers, is based on attractive electrostatic interactions. In addition to poly(phosphobetaines) high attention is especially focused on poly(sulfobetaines) [280–282]. Extensive studies in aqueous solution have shown that the thermoresponsive behavior depends on a variety of parameters, such as concentration, ionic strength, as well as the molecular weight and the spacer length between the charges of the monomers [50–51,283–284]. In particular, the influence of salt ions, which trigger a so-called antipolyelectrolyte effect by screening of the zwitterionic charges, was intensively investigated [285]. As intra- and intermolecular electrostatic interactions are strongly affected by screening, the increase of the ionic strength generally results in a remarkable drop of the phase transition temperature eventually resulting in a complete disappearance of the UCST-type behavior [50,285]. Although zwitterionic polymers display a very sharp phase transition in pure water, their use for biological applications is therefore limited. However, besides zwitterionic compounds, UCST behavior can also be induced in polyelectrolytes via suitable counterions [50]. Analogously, electrostatic interactions dominate the UCST behavior of such charged polymers, which can be manipulated via the hydrophobicity, polarizability, size, and especially the valency of the counterion [50–51]. While Flory et al. investigated the UCST behavior of PAA in the presence of large amounts of NaCl (*c* = 1.245 mol/L) already in 1954, nowadays more and more examples of counterion-induced thermoresponsiveness on charged homopolymers as well as copolymers have been revealed [50–51,286]. In addition to the thermoresponsive behavior of the branched polyelectrolyte polyethyleneimine (PEI) described by Noh et al. [287] in the presence of the halide anions Cl^−^, Br^−^, and I^−-^, respectively, UCST behavior can also be induced in polymeric ionic liquids by the addition of a suitable hydrophobic counterion, such as tetrafluoroborate BF_4_^−^ [288–289]. In both cases, the critical phase transition temperature is strongly affected by the concentration of the polymer, but also by the nature of the anion as well as its concentration. Moreover, UCST behavior induced by BF_4_^−^ was also detected in aqueous solutions of polypeptides, whose side chains contain charged pyridinium or imidazolium functionalities [290–291]. In contrast to single charged counterions, multivalent ions exert a particularly strong influence on polyelectrolytes due to their high charge density [292]. Plamper et al. [293] as well as Zhang et al. [294] demonstrated that in the presence of the triply negatively charged hexacyanoferrate [Fe(CN)_6_]^3−^, polycationic poly(dimethylaminoethyl methacrylate) (PDMAEMA) exhibits UCST behavior in aqueous solution. This is particularly interesting since the polymer initially exhibits LCST behavior in the absence of multivalent ions due to its amphiphilic polymer structure [295–296]. The switchable thermoresponsivity is furthermore complemented by a pH and ionic strength sensitivity [5,297]. The complex phenomenon is referred to as multiresponsivity and has already led to extensive research on PDMAEMA, especially with regard to biomedical applications [5,298–299]. While numerous studies have been performed on free polymer chains in solution, especially focussing on the LCST transition, we have recently shown that an UCST behavior can also be induced in PDMAEMA brushes, thereby generating a novel approach for controllable in situ nanostructuring on surfaces [300–301].

**UCST resulting from biological inspired structures.** In addition to synthetic polymers, thermoresponsivity can also be observed in biological structures and their derivatives. While LCST behavior is more frequently studied in synthetic polymers, the occurrence of UCST behavior predominates in aqueous solutions of proteins [302–303]. The thermoresponse of natural proteins such as β-lactoglobulin, lysozyme or crystallins is based on a complex interplay of various attractive forces such as hydrogen bonding, electrostatic as well as π–π interactions and hydrophobic effects [302,304–305] Several studies show that in the case of natural proteins multivalent ions also have a strong impact on thermoresponsiveness [302–303,305]. Exactly inverse to the behavior of synthetic PDMAEMA, Schreiber and colleagues demonstrate that in the presence of a low concentration of the multivalent salt YCl_3_ (*c* < 2mM), solutions of the globular protein β-lactoglobulin show an unusual LCST behavior [302]. However, when the concentration of the added Y^3+^ ions is increased up to 5 mM, the original UCST-type behavior of the protein is detected again. Isothermal titration calorimetry shows an entropically driven cation binding with a disruption of the highly structured hydration shell of the protein, which governs the LCST behavior. Furthermore, the authors propose that bridging between proteins via the multivalent Y^3+^ ions significantly dominate the unusual LCST behavior. Conversely, however, for synthetic PDMAEMA, it was found that bridging between charged polymer chains via multivalent hexacyanoferrate ions [Fe(CN)_6_]^3−^ leads to an unusual UCST behavior of the polymer [294,300]. Thus, it becomes apparent that even supposedly similar intermolecular interactions such as ionic bridging can lead to different macroscopic outcomes depending on the spatial arrangement of the interacting functional groups.

Driven by the diverse thermoresponsiveness of natural proteins, Quiroz et al. 2015 presented the concept of artificially synthesized disordered proteins (IDPs) [306]. The identification of specific amino acid repeat motifs leading to a desired thermoresponsive outcome of the artificial protein (UCST or LCST) enables encoding a desired phase behavior at the sequential level. The demonstrated platform of, in particular, Pro- and Gly-rich IDPs allows the targeted generation of both LCST and UCST phase transitions, as well as tuning of the phase transition temperature in the range between 20–60 °C within physiologically relevant ionic strength and pH values. Furthermore, the authors show that the fusion of LCST and UCST encoding motifs within one IDP allows the generation of self-assembling structures like micelles. In addition to IDPs, polypeptoids based on an N-substituted glycine backbone, thus biomimetically resembling polypeptides, also obtain high potential for smart biomedical applications [307]. By synthetically varying the charged side chain, Xing et al. recently succeeded in generating both UCST and LCST phase transitions with controllable transition temperature using a single homopolymer polypeptoid backbone [308].

After briefly introducing polymeric building blocks with UCST-type behavior, we will now discuss their use in different topologies in more detail. The analysis of structure–property relationships allows to gain a better understanding of UCST-type phase transitions and therefore reveals beneficial polymeric topologies to be considered for a desired application.

#### Star-shaped architectures

A star-shaped architecture represents a unique class of branched polymers consisting of a central core grafted with several polymer chains, which are forming so-called “arms” stretching into the surrounding solvent. While synthetic approaches for the formation of star polymers as well as their (self-)assembly into complex hierarchical topologies have been extensively studied in current research [309], the influence of branching on the thermoresponsive behavior of polymers is rarely considered [310]. While there are several studies for LCST star polymers (see Table 1), some of them are contradictory, the number of topological studies for UCST star polymers is very limited. However, we will use individual examples to explain the complex impact of the star topology on the thermoresponsive phase transition of polymers and subsequently derive topological advantages of the star architecture.

**Figure 9:**
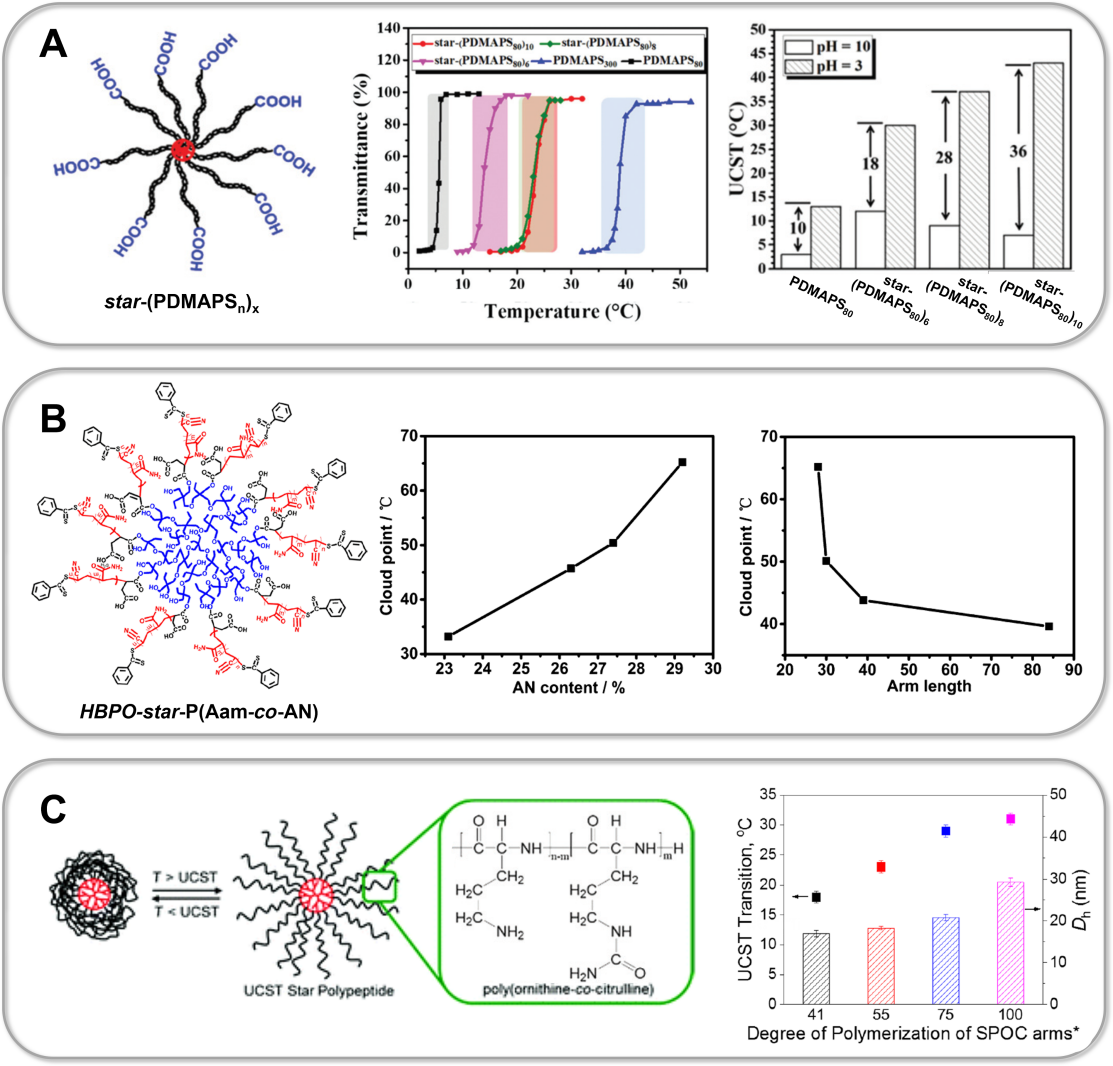
Part A shows the molecular structure of PDMAPS stars synthesized by Li et al. (left) demonstrating tunable UCST-type behavior upon changing the number of arms (middle) as well as changing of the pH value (right) [310]. The responsive behavior of star polymers (PDMAPS_80_)*_x_* in aqueous solution was compared to linear analogs (PDMAPS_80_ and PDMAPS_300_) in order to understand the influence of the star topology. Part B pictures the hyperbranched architecture of P(AAm-*co*-AN)-based star polymers prepared by Qi et al. (left) [313], which demonstrate a characteristic (topologically amplified) dependence of the UCST type cloud point on the AN content of the copolymer as well as the arm length of the stars. Part C shows the UCST type swelling behavior of star polypeptides prepared from poly(ornithine-*co*-citrulline) by Zhou et al., which exhibit arm length dependent UCST transitions [314,317]. Figure 9A was adapted with permission of The Royal Society of Chemistry from [310] (“Synthesis of star-shaped polyzwitterions with adjustable UCST and fast responsiveness by a facile RAFT polymerization” by Z. Li et al., *Polym. Chem.*, vol. 11, issue 18, © 2020); permission conveyed through Copyright Clearing Center, Inc. This content is not subjected to CC BY 4.0. Figure 9B was adapted with permission from [313]. Copyright 2018 American Chemical Society. This content is not subject to CC BY 4.0. Figure 9C (left) was adapted with permission of The Royal Society of Chemistry from [314] (“Enzymatically degradable star polypeptides with tunable UCST transitions in solution and within layer-by-layer films” by Q. Zhou et al., *Polym. Chem.*, vol. 9, issue 40, © 2018); permission conveyed through Copyright Clearing Center, Inc. This content is not subjected to CC BY 4.0. Figure 9C (right) was adapted with permission from [317]. Copyright 2019 American Chemical Society. This content is not subject to CC BY 4.0.

In a recent study Li et al. synthesized zwitterionic star shaped polymers showing a dual pH- and UCST-type thermoresponsiveness in aqueous solution [310]. Using an “arm-first” approach they prepolymerized PDMAPS as a macroRAFT agent. In a second RAFT polymerization the PDMAPS arms were subsequently assembled into a star architecture via the addition of a crosslinker. Li and colleagues thus succeeded in preparing 6-, 8- and 10-arm star polymers (PDMAPS_80_)*_x_*_,_ whose thermoresponsive behavior was thoroughly compared to linear analogues ((PDMAPS_80_) and (PDMAPS_300_)). Turbidimetry at pH 7 shows an increase in the UCST-type transition temperature of the 6-arm to the 8-arm starPDMAPS from 16 °C to 23 °C (Figure 9, part A). The authors attribute this shift to an increased molecular weight of the polymers, thus resulting in enhanced intermolecular electrostatic attraction. This is consistent with studies on linear polymer chains in solution, which generally show an increase in the phase transition temperature of UCST-type polymers with increasing molecular weight due to a decreasing entropy of mixing [51,311]. Interestingly, the phase transition temperature of the 8-arm star polymer (*T*_c_ (PDMAPS_80_)_8_ = 23 °C) is significantly lower than that of linear PDMAPS_300_ (*T*_c_ (PDMAPS_300_) = 39 °C), although the authors claim both polymers have a comparable molecular weight of ≈67 kDa [310]. Since DLS measurements confirm a lower hydrodynamic radius *D*_h_ and thus a denser arrangement of polymer chains in the star architecture, one might speculate that the *M*_n_ of the star polymer obtained via GPC measurements is still significantly underestimated. However, the increased local segment density surprisingly does not enhance attractive intermolecular interactions, thus leading to a decreased UCST of the star polymer. This example demonstrates very clearly that the influence of a polymer's architecture on its thermoresponsiveness is usually not easy to predict and should be carefully studied for each individual system. Moreover, the very sharp and fast phase transition of the PDMAPS star polymers even at high molecular weight is particularly noteworthy (Figure 9, part A). This topological advantage could be a promising strategy to avoid a broadening of the phase transition window with increasing molecular weight as observed for linear zwitterionic polymers by Shih et al. [311]. Furthermore, the relatively broad phase transition of linear UCST polymers, in contrast to well-known LCST systems, is still an urgent issue, which needs to be addressed in order to develop fast switching systems for novel actuator or sensor applications [38,51,311–312]. In addition, Li and co-workers were able to exploit the high density of functional groups within the star topology to tune the thermoresponsive behavior with a second external trigger. Carboxylic end-groups allowed the gradual increase of the UCST in the pH range from 3 to 10 by 18 °C for the 6-arm star polymer. By increasing the number of arms an even larger shift of up to 36 °C was achieved for the 10-arm star polymer, whereas for the linear polymer PDMAPS_80 _*T*_c_ could only be tuned within a window of 10 °C (Figure 9, part A). In a comparable manner, Qi et al. were able to induce a so-called amplification effect using a hyperbranched thermoresponsive copolymer [313]. Herein RAFT polymerization of P(AAm-*co*-AN)-arms onto a branched hydrophobic core yielded the characteristic star architecture pictured in Figure 9, part B. Unlike charged zwitterionic polymers, hydrogen bonds control the thermoresponsive behavior of the neutral copolymer P(AAm-*co*-AN). Consequently, the system is less sensitive to ionic strength and shows a reversible, sharp phase transition in water as well as electrolyte solution. Variation of the AN content within the copolymer enables tuning of the cloud point, which is a feature also known from linear analogues [94]. Interestingly, the branched architecture showed a large shift of the UCST-type cloud point from 33 °C to ≈65 °C by very slightly increasing the AN fraction from 23 to 29%. Studies on the linear copolymer show a lower tunability and illustrate that a hyperbranched architecture can significantly enrich the thermoresponsive behavior [94,275,313]. To further study the effect of molecular architecture, Qi et al. synthesized a set of star polymers containing a constant AN fraction of 29% with variable arm length. Increasing the degree of polymerization of the arms from 28 to 84 leads to a decrease in the cloud point from 65.2 °C to 39.6 °C [313]. This represents an inverse dependence compared to linear P(AAm-*co*-AN), where an increase in *T*_c_ is observed with an increase in molecular weight [274–275]. The authors propose that this inverse trend is based on a pronounced hydration of long polymer arms, which leads to stretching into the surrounding solution away from the hydrophobic core. One might speculate as well that the sterically restricted geometry of the star polymer hampers the formation of intermolecular hydrogen bonds, which are crucial for the UCST-type response. However, Zhou et al. demonstrate that in the case of star-shaped polypeptides (star-poly(ʟ-ornithin-*co*-ʟ-citrullin) (SPOC), the increase in arm length from ≈60 kDa to ≈100 kDa results in an increase in the UCST-type phase transition temperature from 18 °C to 31 °C [314] (Figure 9, part C). Although analogous to the star-shaped P(PAAm-*co*-AN) of Qi et al., the UCST behavior is based on hydrogen bonding, the steric confinement of the polypeptide chains does not seem to hamper attractive intermolecular interactions in this case [313]. Moreover, it is demonstrated that the high local concentration of functional groups and therefore strong polymer–polymer interactions lead to a higher *T*_c_ of the star polypeptide compared to a linear analogue with matching polymer chain length and similar composition [314]. Interestingly, the dendritic topology of the star polypeptide revealed a very low concentration dependency of the phase transition temperature. While a *T*_c_ shift of more than 10 °C was detected for linear ureido-based polymers when the concentration was increased from 0.5 mg/mL to 2.5 mg/mL [315], the *T*_c_ varied only by a few degrees Celsius for the star polypeptides (<3 °C) [314]. The phase transition itself occurs within a remarkably small temperature window (<5 °C). Due to the conformational restriction of the polypeptide in the star architecture, the formation of secondary structures can be strongly inhibited. This contrasts with the increasing formation of β-sheets during multiple heating/cooling cycles of linear polypeptides, which can lead to irreversible precipitation [314,316]. In addition, Zhou et al. also succeeded in assembling star polypeptides via a layer-by-layer technique using hydrogen bond-forming tannic acid into a functional surface coating [314,317]. Importantly, for all thin films of the star polypeptides the UCST transition shifted to much higher temperatures than in solution. In particular, the *T*_c_ of the star-polypeptide SPOC_55_-96 in solution increased from around 24 °C to around 44 °C, when assembled into a thin film with tannic acid via hydrogen bonding interactions. Furthermore, ellipsometric studies of the films show that in this physically crosslinked geometry, a much broader and thus slower phase transition of the star polypeptides occurs. This again illustrates that the spatial arrangement of thermoresponsive materials exerts a strong influence on the phase transition. This was also shown by Willcock and co-workers, who investigated the influence of branching on zwitterionic PDMAPS with molecular weights ranging from 5 to 500 kDa [318]. The introduction of bifunctional monomers leading to additional side chains of the zwitterionic polymers results in vastly reduced transition temperatures compared to their linear analogs. The shift of *T*_c_ to lower temperatures often results in a complete disappearance of the UCST behavior in the measurable temperature range of 0 to 100 °C. The results are in accordance with studies on zwitterionic hydrogels where a strong decrease of UCST-type *T*_c_ is observed with increasing crosslinking density [55,318–320]. Interestingly, Willcock and colleagues propose a model of reduced effective molar mass of branched polymer chains in order to describe the influence of branching on their thermoresponsive behavior. The thermodynamic theory of polymers in solution states that in general the UCST increases with increasing molecular weight of the chains due to a decrease of the entropy of mixing [51]. Thus, an effectively lower chain length for branched polymers could well explain a significant decrease in UCST with increasing branching. However, the very simple model neglects the steric constraint of the branched polymer chains, which can lead to a dramatic change in the attractive polymer–polymer interactions governing the UCST behavior.

This phenomenon is also illustrated by Zhang et al. on a star-shaped copolymer consisting of a zwitterionic as well as a cationic block grafted on a hydrophobic cyclodextrine core [321]. Due to the UCST behavior of the zwitterionic polymer, an increase in temperature causes the zwitterionic block to collapse around the cationic segment, which results in the formation of an outer corona of sticky patches. While this particular behavior occurs in all 12-arm star copolymers, an UCST behavior of the zwitterionic block is completely suppressed due to steric constraints, when the grafting density and thus the number of arms is increased to 17. Interestingly, the high local density of polymer segments within the star architecture also influences screening effects of the zwitterionic block by added salts. Known as the antipolyelectrolyte effect, salt ions can shield charges in zwitterionic polymers. This leads to an increase in solubility and thus a sharp drop in UCST until the thermoresponsive behavior eventually disappears completely even at relatively low ionic strength [50,281,322]. This phenomenon still hinders the wide use of zwitterionic polymers in physiological media for biomedical applications, although they have long been known for their sharp and reversible UCST-type behavior in water. However, the high local density of polymer segments within the star polymer leads to a limitation of shielding effects, resulting in a salt tolerance of up to 20 mM NaCl, which is more than 10 times higher than that of linear zwitterionic polymers (*c* ≈ 0.7–2 mM) [321]. Furthermore, the association behavior of the star polymers in aqueous solution could be specifically influenced via the grafting density as well as the arm length. While association processes were strongly suppressed for high grafting densities, increasingly large spherical aggregates were detected below the critical phase transition temperature (*T* < UCST) with increasing arm length of the star polymers. Benefits and limitations of star-shaped topologies on the UCST behavior of polymers are summarized in Table 2.

**Table 2:** Benefits and limitations arising from a star topology on the thermoresponsiveness of UCST-type polymers.

Benefits	Limitations

• Often very sharp phase transitions (small temperature window, in which transitions takes place). • Often low concentration dependence of the transition temperature. • High density of functional groups (beneficial for the implementation of a secondary external trigger). • Grafting density and arm length of the star architecture can be used to control *T*_c_ as well as the aggregation behavior. • Impact of the antipolyelectrolyte effect reduced for zwitterionic star polymers (increased salt tolerance). • Excellent rheological properties (low viscosity).	*• T*_c_ can drop significantly if steric restrictions of the star topology hamper attractive polymer–polymer interactions. • *T*_c_ is strongly dependent on the grafting density and the arm length of the star polymer, but is difficult to predict (general trends may vary depending on the chemical composition of the star polymer).

#### Block copolymers forming micellar structures

Polymers with LCST or UCST behavior can undergo a single-phase transition upon variation of temperature (coil-to-globule transition), which is often attributed with switching between a hydrophilic and a hydrophobic physicochemical state [51–52]. However, if several thermoresponsive segments are linked to form a block copolymer, sequential transitions between more than two states can be obtained. Often, two blocks of the same (LCST–LCST, UCST–UCST) or different responsivities (UCST–LCST) are linked to form a so-called dual thermoresponsive block copolymer [55,323–325]. Sequential transitions of the individual blocks allow reversible switching between hydrophilic, amphiphilic and hydrophobic states of the copolymer. In aqueous solutions, the block copolymer can consequently be present either in fully dissolved state (hydrophilic) as well as within self-assembled micelles (amphiphilic) or as macroscopic aggregates (hydrophobic). Moreover, when generating multiblock copolymers with more than two different thermoresponsive segments, an increasingly complex thermoresponsiveness with an increasing number of structural phase transitions can be obtained. For a triblock copolymer, 12 different structural modes should be conceivable due to different arrangement possibilities of the UCST/LCST blocks [323–324]. Sugihara et al. succeeded in preparing a LCST–LCST–LCST triblock copolymer showing a reversible multistage morphology transformation from sol (*T* < 20 °C) to individual micelles (20 °C < *T* < 41 °C) towards physical gelation (41 °C < *T* < 61 °C) and precipitation (*T* > 64 °C) [326]. However, even in the case of a diblock copolymer, the thermoresponsive phase transitions are strongly influenced by a variety of parameters such as length and interaction of the different blocks, polymer concentration, but also by the selected solvent and added salt ions [325]. This results in a large variety of self-assembly scenarios, which gives rise to a broad range of micellar structures. In the following, we will limit ourselves to dual thermoresponsive block copolymers and refer the interested reader to further literature on multi-block copolymers [323].

**Figure 10:**
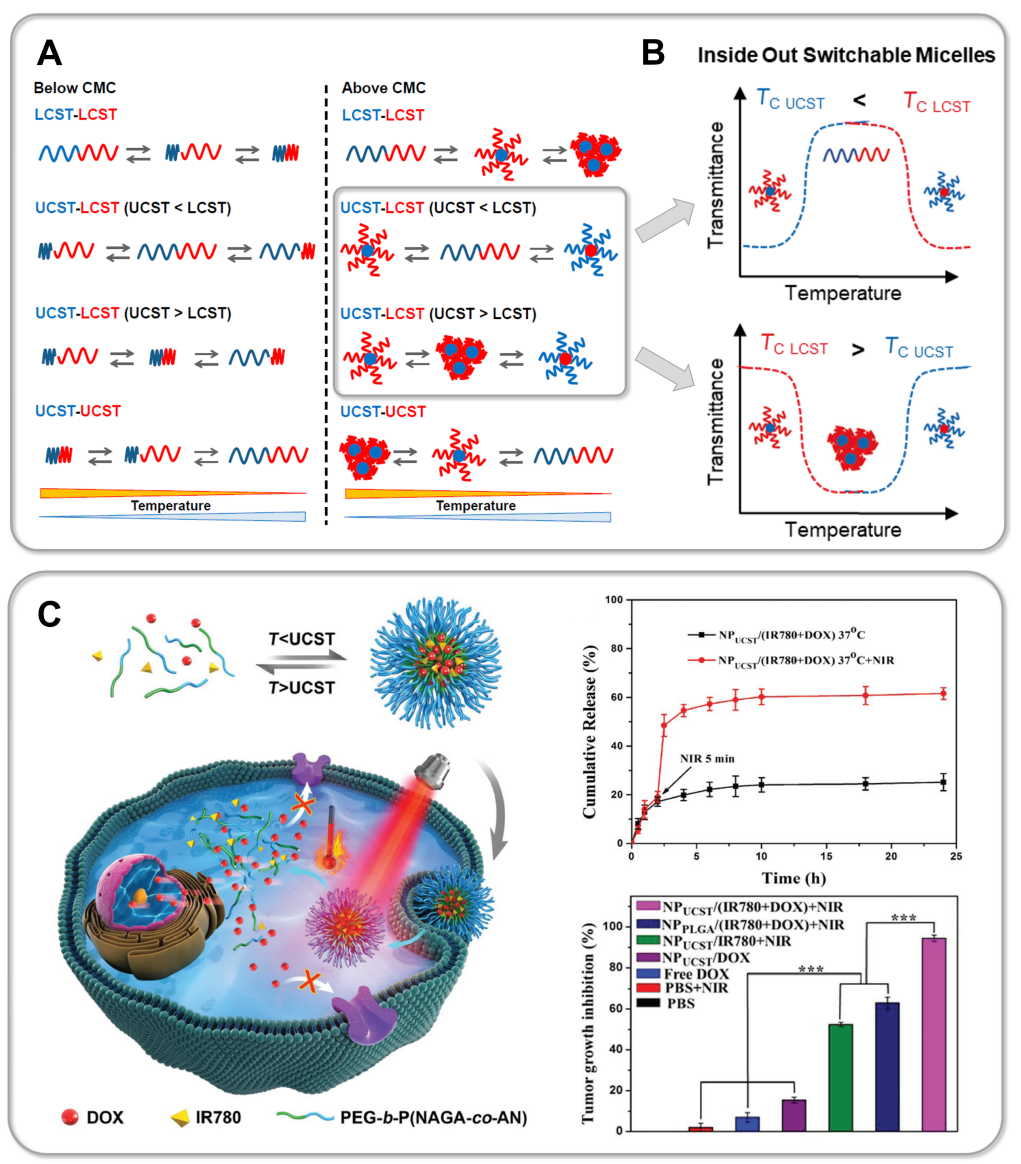
Part A contains a schematic demonstration of conformational transitions of dual-thermoresponsive block copolymers above and below the critical micelle concentration (CMC) adapted from Kotsuchibashi et al. [324]. The temperature-dependent self-assembly of UCST–LCST block copolymers into inside-out switchable micelles, whose characteristic transmittance curves are schematically pictured in part B, is of particular interest. In part C, the use of UCST block copolymers as smart nanocarriers for drug delivery is exemplified by the work of Deng et al. [330]. The UCST-based disassembly of micelles triggered by near-infrared radiation onto the internalized photosensitizer IR780, which causes the on demand drug release of doxorubicin hydrochloride (DOX) into the cancer cell, is shown schematically on the left. Based on the successful application of the NIR trigger leading to a burst release of DOX (upper right), excellent tumor growth inhibition is registered within in vivo studies using a mouse model (bottom right). Figure 10A was adapted from [324] (© 2016 Kotsuchibashi, Y. et al., published by MDPI, Basel, Switzerland, distributed under the terms of the Creative Commons Attribution 4.0 International License, https://creativecommons.org/licenses/by/4.0). Figure 10B was redrawn from [55]. Figure 10C was adapted from [330], Deng et al., “Let There be Light: Polymeric Micelles with Upper Critical Solution Temperature as Light-Triggered Heat Nanogenerators for Combating Drug-Resistant Cancer”, S*mall,* with permission from John Wiley and Sons. Copyright © 2018 WILEY-VCH Verlag GmbH & Co. KGaA, Weinheim. This content is not subject to CC BY 4.0.

According to the segments they contain, dual thermoresponsive block copolymers can be classified into four different categories [55]. If similar responsive blocks are used, LCST–LCST as well as UCST–UCST systems can be generated. If different types of segments are combined, LCST–UCST copolymers are created in which either LCST > UCST or LCST < UCST is present. In all systems, sequential phase transitions of the individual blocks occur, resulting in a conformational change of the entire block copolymer. Above and below the critical micelle concentration (CMC), different structural scenarios arise according to the composition of the copolymer, which were summarized schematically by Kotsuchibashi et al. (Figure 10, part A) [324]. While multiple LCST–LCST systems have already been studied, often using at least one PNIPAAm block, to the best of our knowledge there has been no report of a UCST–UCST system so far [55,324]. In addition to the still significantly lower number of available UCST systems, this is probably also due to the more complex phase behavior of UCST polymers. Since the phase behavior is determined by oriented intra- and intermolecular interactions, gaining precise control over distinguishable critical transition temperatures for each block is particularly difficult. Even in UCST–LCST systems, the selection and control of the UCST block is usually more challenging compared to the LCST counterpart [325]. Despite all obstacles, UCST–LCST systems are particularly interesting because they offer the self-assembly of so-called inside-out switchable micelles. With increasing temperature, a copolymer of the type UCST < LCST can transform from a micellar structure with the UCST segment in the core, via fully dissolved unimers, to a micelle with the LCST block in the solvophobic core. Turbidimetry can be used to measure the high optical transmittance of the dissolved unimers at intermediate temperatures, as well as the turbidity of the solution due to the micelle structures at low (*T* < UCST) and high temperatures (*T* > LCST) (Figure 10, part B). Similarly, copolymers of the UCST > LCST type also self-assemble into switchable micelles, though connected via an aggregated state at intermediate temperatures [55,325]. Thus, in both scenarios, without varying the chemical composition of the copolymer, a simple thermal stimulus can be exploited to provide different types of microdomains in the core as well as a switchable micelle corona interacting with the solvent [280]. The unique properties of such dual responsive block copolymers therefore have a great potential for application in the field of biosensing, smart drug delivery, emulsification systems as well as in smart rheology [55,325]. For the first time such an UCST–LCST copolymer was synthesized by Arotҫaréna et al. in 2002 [327] fusing an LCST exhibiting nonionic PNIPAAm block and a zwitterionic poly(*N,N*-dimethyl-*N*-(3-(methacrylamido)propyl)ammoniopropane sulfonate) (PSPP) block, showing UCST type behavior. While the PNIPAAm block exhibited a constant *T*_c LCST_ of around 32 °C, which differed only slightly from the homopolymer, varying the length of the zwitterionic block shifted the *T*_c UCST_ between 9 and 19 °C. However, slightly broader phase transitions of the different blocks in the copolymer were observed compared to the homopolymers. The characteristic inside-out switching of the self-assembled micelles was detected via viscosity and fluorescence measurements as well as ^1^H NMR and turbidimetry. In a recent feature article, Papadakis and colleagues also profoundly discuss diblock copolymers consisting of a nonionic PNIPAAm and a zwitterionic poly(sulfobetaine) block [325]. The comparison of the cloud points *T_c_* (LCST/UCST) of the homopolymers PNIPAAm_200_ and PSPP_430_ with the block copolymer PSPP_430_-PNIPAAm_200_ showed interesting variations due to the respective nature of the thermoresponsive block. While the *T*_c LCST_ of the block copolymer of 32.3 ± 0.5 °C is not changed, a decrease in the *T*_c UCST_ of the copolymer by about 8 K to 21.2 ± 0.5 °C is observed [328]. Whereas the UCST transition is strongly affected by the polymer architecture and the presence of the LCST block, conversely the nonionic PNIPAAm segment behaves rather unaffected by the UCST type block. This illustrates that the double thermoresponsive behavior of a block copolymer is not a simple superposition of the phase behavior of the fused homopolymers [325]. This should be taken into account when designing novel structures for future smart applications. Vishnevetskay et al. also demonstrate that only the UCST behavior of a PSPP–PNIPAAm block copolymer was dependent on ionic strength (addition of NaBr) and polymer concentration, while the LCST transition was unchanged [328]. This is consistent with the original findings of Arotҫaréna et al. as well as further studies on block copolymers of varying segment length and structural derivatives of PNIPAAm and PSPP [325,327]. Usually, a decrease in the *T*_c UCST_ of the zwitterionic block is observed with increasing ionic strength, as well as a decreasing length of the UCST block, which is in accordance with the well-known behavior of zwitterionic homopolymers. Moreover, Vishnevetskay et al. recently succeeded in exploiting the high sensitivity of the UCST block to secondary external triggers for selectively switching between two self-assembly routes of the thermoresponsive block copolymer [322]. By combining a sufficiently long zwitterionic block of poly(4-((3-methacrylamidopropyl)dimethylammonio)butane-1-sulfonate) (PSBP) with a PNIPAAm segment, a block copolymer was generated (PSBP_245_-b-PNIPAAm_105_), in which the *T*_c UCST_ is shifted above the *T*_c LCST_ under salt-free conditions (UCST > LCST). Consequently, thermoresponsive switching of the inside-out micelles proceeds via an aggregated intermediate state. Upon addition of NaBr, the *T*_c UCST_ continuously decreases in contrast to the *T*_c LCST_. When a critical concentration of 16 mM salt is exceeded, the *T*_c UCST_ drops below the *T*_c LCST_ (UCST < LCST). The assembly behavior of the block copolymer now changes and the transition between the inverse micelles occurs via fully solubilized unimers (Figure 10, part B). The sophisticated use of this orthogonal trigger superimposing the thermoresponse of the block copolymer offers promising opportunities especially in the field of smart carrier systems as well as emulsifiers. For the development of novel carriers, it is also interesting to note that the properties of the solvophobic micelle core can vary greatly depending on whether it is formed by the phase-separated UCST block (*T* < UCST, LCST) or the LCST block (*T* > LCST, UCST). In the case of a block copolymer synthesized by Hildebrand et al. the zwitterionic UCST block in the collapsed micelle core still had a high polarity and therefore contained large amounts of water, while the collapsed PNIPAAm core, present at high temperatures, was highly dehydrated [280]. The different microdomains thus formed in the micelle cores could be potentially used for selective solubilization as well as a triggered release of certain compounds, such as drugs. Furthermore, Cummings et al. were able to covalently anchor an analogous copolymer to the enzyme chymotropsin via surface-initiated RAFT polymerization [329]. The structural collapse of the zwitterionic UCST block at low temperatures, as well as of the PNIPAAm LCST block at high temperatures were successfully exploited to modulate the substrate affinity of the enzyme within the bioconjugate. The anchored polymer shell led to an increased stability of the enzyme by variation of the temperature, pH, and proteatic degradation, resulting in a stable bioconjugate for more than 8 h at pH 1.0 in the presence of stomach protease.

Recently, the discovery of novel UCST polymers has led to advancements in thermoresponsive block copolymers [331–332]. Zhang et al. [333], Käfer et al. [334], and Zhou et al. [335] succeeded in embedding the nonionic UCST-type copolymer P(AAm-*co*-AN) into dual thermoresponsive (UCST–LCST) systems, which were complemented either by a PDMAEMA, PEG or PNIPAAm LCST block. In contrast to the use of zwitterionic polymers, a reversible, sharp UCST phase transition of the P(AAm-*co*-AN) block could be detected in pure water as well as buffer solutions of elevated ionic strength. The transition temperature could be controlled by the AN content of the copolymer. The UCST behavior very clearly reflected the known responsive behavior of pure P(AAm-*co*-AN) and was only slightly affected by the corresponding LCST block. In all examples, characteristic self-assembled in-side out micelles were detected via turbidity measurements as well as dynamic light scattering (DLS), and AFM. According to the relative position of UCST and LCST, a transition between the inverted micelles via fully solubilized unimers was detected for copolymers with a PNIPAAm or a PDMAEMA block, respectively (UCST < LCST), whereas Käfer et al. observed an aggregated transition state using a PEG block (UCST > LCST) [334].

The development of dual thermoresponsive block copolymers has attracted considerable academic interest in recent years due to the complexity of the phase transitions and the obtainable self-assembled structures. However, copolymers containing an UCST block as the only responsive unit have already been characterized more comprehensively and are currently leading towards smart applications, especially in the field of triggered drug release systems for cancer therapy. Based on the pioneering work of Li et al. [336] in 2015, in recent years (2016–2020) several research groups succeeded in developing the first UCST-based drug delivery nanocontainers showing excellent results in their application in vitro as well as within in vivo studies [272,330,337–341]. Due to their robust phase behavior under biologically relevant conditions (pH, ionic strength, etc.), block copolymers with nonionic UCST segments of P(AAm-AN) [272,336–341] or PNAGA [314] exhibiting a transition temperature of ≈43 °C were used in all studies. The major advantage of these novel systems, compared to their LCST-based counterparts, is the temporally and spatially very well controllable supply of heat needed for the responsive phase transition of the UCST polymers, thus leading to an on-demand release of the active drug molecules. In addition to the chemotherapeutic agent, usually doxorubicin, a photothermal agent such as cyanine dye IR780 is incorporated into the micelles. After accumulation of the micelles in the tumor tissue via the EPR effect, heat can be generated by temporal, local irradiation of an NIR laser (often λ = 808 nm, *t* < 5 min). This local hyperthermia can already produce enhanced tumor necrosis, it also leads to a phase transition of the UCST polymer block, which results in a disassembly of the micelles and a triggered local release of the drug (Figure 10, part C). Low leakage rates of the drug in the micellar "off-state" as well as rapid and often >70% on-demand release demonstrate the excellent properties of these nanocarriers, which in some cases are superior compared to well-known LCST-based systems [336,338]. In vivo studies in mouse models generally demonstrate both high accumulation of UCST-type micelles in the tumor tissue as well as excellent anticancer efficiency of the combined photothermal chemotherapy, which is often reflected in a significant reduction of cell viability and tumor size. The modular structure of the UCST block copolymers allowed Zhan et al. [338] as well as Yang et al. [339] to incorporate another responsive block with responsiveness towards reactive oxygen species or reduced pH in the tumor tissue, which lead to synergistic anticancer efficiencies. Furthermore, Yang et al. [341] succeeded in integrating the anticancer agent doxorubicin hydrochlorid (DOX) in the form of a pro-drug in the UCST block copolymer via a thermolabile linker, thus minimizing unwanted drug leakage below the UCST.

In this chapter we analyzed the behavior of block copolymers, containing at least one UCST-type block supplemented by other responsive or nonresponsive segments with particular focus on their temperature dependent self-assembly into micelles. Finally, in analogy to star polymers (Table 2), we summarize benefits and limitations of this topology and the resulting UCST-based thermoresponsive behavior (Table 3).

**Table 3:** Benefits and limitations arising from UCST based block copolymers and their assembly into switchable micelles.

Benefits	Limitations

• Often, steric restrictions in self-assembled block copolymer micelles are smaller than in covalently fused star polymers, therefore the UCST block usually resembles more strongly the phase behavior of the linear homopolymer. However, a shift in *T*_c UCST_ caused by the attached copolymer strongly depends on the UCST mechanism (ionic, non-ionic). • Dual thermoresponsive block copolymers (UCST-LCST) offer the self-assembly into in inside-out switchable micelles containing different microdomains in one polymer, which carries high potential for smart nanocarriers. • Despite of the thermoresponsive transition, precipitation can be strongly suppressed, especially in contrast to homopolymers. • First successful in vivo studies of UCST block copolymers as drug delivery systems for cancer therapy. Temporally, localized heat can be generated easily, which provides an advantage over LCST based systems requiring cooling. Combined photo-thermal and chemotherapy yields synergistic effects. • The modular structure of block copolymers can be extended to further blocks with specific properties.	• In particular, dually thermoresponsive block copolymers can have very complex phase transitions/assembly patterns. • Often the thermoresponsive behavior of the UCST segment is more strongly influenced by other segments than vice versa

### UCST polymers grafted to flat substrates

To apply thermoresponsive polymers for an intelligent control of surface properties the polymer chains need to be anchored to a substrate material. Thus, the temperature behavior of such systems is no longer just an interplay of the polymer chemistry of free chains and the surrounding solvent, but is largely determined by the assembly of the chains as well as the interaction between the polymer and the support material [124,342]. While in numerous reviews about the behavior of grafted LCST polymers it is shown that the grafting architecture not only influences the value of the critical phase transition temperature but often broadens the responsive transition as well, there are only few experimental studies on grafted UCST polymers so far. In the following, we would like to summarize the existing studies and draw conclusions for future applications of grafted UCST polymers. However, we would also like to point out that many topological effects, although some of them are very promising, are not well understood and exploited in applications yet.

**Figure 11:**
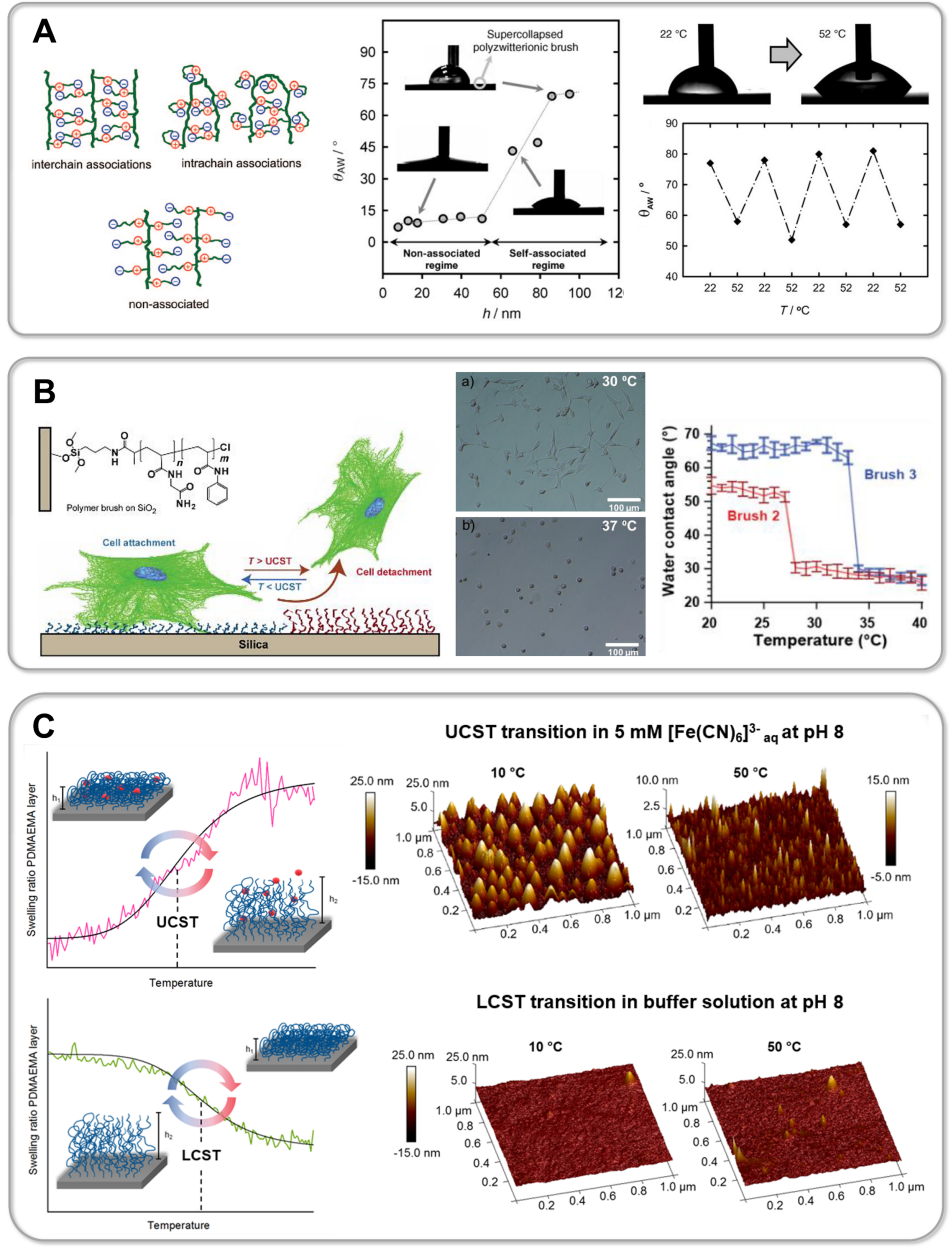
Part A pictures zwitterionic brushes grafted from silicon substrates obtaining a nonassociated, hydrophilic state at small brush thicknesses compared to thick brushes exhibiting hydrophobic surface properties due to a supercollapsed state based on inter- and intrachain associations (left). Temperature-dependent contact angle measurements of supercollapsed zwitterionic brushes by Azzaroni et al. (right) [90]. Part B shows the UCST-mediated cell adhesion of a silicon substrate coated with PNAGA brushes from Xue et al. [346]. Microscopic images herein monitor the cell-adhesive state at 30 °C (*T* < UCST) and the cell-repulsive state obtained at an elevated temperature of 37 °C (*T* > UCST) (right). Part C demonstrates the switchable character of PDMAEMA brushes prepared by Flemming et al. [300–301], which exhibit a LCST transition in presence of monovalent salts (bottom), whereas an UCST transition can be induced in the presence of multivalent ions like [Fe(CN)_6_]^3−^ (top). Spectroscopic in situ ellipsometry monitors the temperature-dependent swelling of the brushes (left), while in situ AFM images capture the surface properties of the brushes (right). Homogeneous brush surfaces are observed during the LCST transition, while the induced UCST transition is characterized by the appearance of pinned micelles with entrapped multivalent ions. Figure 11A was adapted from [90], O. Azzaroni et al., “UCST Wetting Transitions of Polyzwitterionic Brushes Driven by Self-Association”, *Angew. Chem. Int. Ed.*, with permission from John Wiley and Sons. Copyright © 2006 WILEY-VCH Verlag GmbH & Co. KGaA, Weinheim. This content is not subject to CC BY 4.0. Figure 11B was adapted with permission of The Royal Society of Chemistry from [346] (“Upper critical solution temperature thermo-responsive polymer brushes and a mechanism for controlled cell attachment” by X. Xue et al., *J. Mater. Chem. B*, vol. 5, issue 25, © 2017); permission conveyed through Copyright Clearing Center, Inc. This content is not subjected to CC BY 4.0. Figure 11C (left) was adapted with permission from [300]. Copyright 2020 American Chemical Society. This content is not subject to CC BY 4.0. Figure 11C (right) was adapted with permission from [301]. Copyright 2020 American Chemical Society. This content is not subject to CC BY 4.0.

The first polymer brush to demonstrate UCST behavior was fabricated in 2006 by Azzaroni et al. [90], using surface-initiated ATRP, homogeneous and patterned zwitterionic brushes on gold and silicon substrates were synthesized from [2-(methacryloyloxy)ethyl]dimethyl(3-sulfopropyl)ammonium hydroxide (MEDSAH). While extensive studies of zwitterionic polymers in bulk as well as in solution were available at that time [343], the fundamental work of Azzaroni et al. provides the first insight into how these polymers behave within dense layers assembled on a surface [90]. The preparation of brushes of different layer thicknesses as well as their extensive characterization by means of contact angle measurements, Auger electron spectroscopy and AFM allows for the first time to identify molecular self-assembly processes within the brush. Accompanied by a sharp increase in the contact angle against water, Azzaroni and colleagues show that brushes with low film thickness (*d* < 50 nm) exist within a nonassociated state, while at high film thicknesses (up to 100 nm) inter- and intrachain ionic bridges are formed within an avalanche-type association process resulting in a supercollapsed state of the brush (Figure 11, part A) [90]. The thickness-dependent association states resulted in hydrophilic surfaces with an advancing water contact angle of about θ_AW_ ≈ 12° for thin brushes, while for thick brushes θ_AW_ increased up to ≈79° (at *d* = 180 nm) and thus a pronounced hydrophobicity was detected. AFM measurements on patterned brushes confirmed that strong swelling and thus hydration of thin brushes occurs (*d*_air_ = 50 nm < *h*_water_ = 150 nm), while supercollapsed thick films hardly showed any swelling behavior with the film thickness being effectively constant both in air (*d* = 90 nm) and under water (*h* = 96 nm). Derived from theoretical studies, describing a high sensitivity of the electrostatic microenvironment of zwitterionic polymers to their conformation [344], Azzaroni et al. suggest that, in particular, the increase in chain length and thus the increasing number of potential ion pairings per grafting site enables the transition of the brush to a supercollapsed state [90]. However, further studies by Cheng et al. in 2008 show that additionally to a sufficient molecular weight the grafting density of the brush is a key factor [345]. For densely grafted polymer brushes the critical layer thickness for a supercollapsed state was relatively low, whereas for brushes with low grafting density higher layer thicknesses were required.

Temperature-dependent contact angle measurements, performed by Azzaroni et al., confirmed for the first time the UCST-based hydrophobic-to-hydrophilic transition of zwitterionic supercollapsed brushes (*d* = 180 nm) in which θ_AW_ decreased from 79° to 58° during a temperature increase from 22 °C to 52 °C (Figure 11, part A) [90]. Repeated temperature cycling indicated that this UCST transition is completely reversible and the magnitude of the effect is comparable to the well-established LCST transition of PNIPAAm brushes [347]. However, it is interesting to note that the UCST-type transition temperature of the zwitterionic brushes of ≈40 to 50 °C is significantly higher than that of comparable free polymer chains in solution (≈30 °C) [343]. The authors suggest that this is caused by trace impurities (<0.5 mol %) of inorganic salts arising from ATRP polymerization. However, based on the meanwile extensively studied antipolyelectrolyte effect [50], it is reasonable to assume that the presence of monovalent salts leads to a decrease in UCST. Therefore, we assume that the significantly increased transition temperature is due to the confined arrangement of the polymer chains within the brush structure thus promoting ion pair formation. Cheng et al. also observed similar high transition temperatures of zwitterionic brushes [345]. They also demonstrate that during the UCST transition densely grafted brushes show a low switching amplitude of ≈10–15° while a change in contact angle of up to 35° was measured for diluted brushes. Thus, the brush conformation determines not only the phase transition temperature itself, but also the macroscopic switching efficiency. A similarly large UCST-based switching effect was obtained by Yuan et al. using quaternized PDMAEMA brushes grafted from a graphene oxide sheet [348]. Upon increasing the temperature from 5 °C to 60 °C, a decrease in the static water contact angle (θ_SW_) from ≈68° to ≈43° (∆θ_SW_ ≈ 25°) was detected. Interestingly, comparable nonquaternized PDMAEMA brushes, inherently exhibiting LCST behavior, showed an inversed switching effect with a slightly lower switching amplitude of ∆θ_SW_ ≈ 20°. Recently, Chen et al. (2020) reported the adaptive wettability of an UCST-based surface with an extremely high switching amplitude and a very fast response rate [349]. Switching from a hydrophobic state at 30 °C with a θ_SW_ of ≈103° to a hydrophilic surface at 80 °C with a θ_SW_ of ≈60° (∆θ_SW_ ≈ −43°) occurred with a maximum response rate of ≈11° s^−1^. The reversible switching behavior is superior in all parameters to those of known LCST-based PNIPAAm systems. However, it should be noted that the developed interface is a multicomponent system. Chen et al. used a porous aluminum oxide substrate with 100 nm sized nanopores, which was coated with both the UCST copolymer P(AAm-*co*-AN) and a fluorosilane (1*H,*1*H,*2*H,*2*H*-perfluorooctyltriethoxysilane; PFOTES). Based on reference studies on flat substrates as well as coatings containing only the single component (P(AAM-*co*-AN) or PFOTES), it became evident that the excellent UCST-based switching of the multicomponent system arises from synergistic effects. In particular, porosity greatly improved the UCST-based switching efficiency.

In addition to successfully adapting the wettability of a surface, Shen et al. [350] were able to generate a temperature-dependent ion permeability for energy storage devices containing nonaqueous electrolytes. For this purpose, a graphene oxide sheet was coated with zwitterionic poly(sulfobetaine) brushes using surface-initiated ATRP. While the Li ion flux is almost unimpeded below the UCST with the brushes obtaining a collapsed conformation, at elevated temperatures the unfolding zwitterionic chains begin to interact more and more with the surrounding electrolyte. The ion flux and the specific capacity for Li ions is reversibly reduced by more than 50%, when the temperature is increased from 20 °C to 80 °C. The responsive mechanism thus effectively prevents a thermal runaway, which is still a fundamental problem in the use of lithium ion batteries. In addition to the application of grafted UCST polymers in electronic systems as well as within switchable gating membranes [351], in recent years there has been a growing interest to develop UCST-based surfaces for controlled cell adhesion within cell manufacturing and regenerative medicine [346,352–353]. Xue et al. succeeded in preparing two different types of UCST-based thermoresponsive surfaces for controlled cell attachment between 2017 and 2018 [346,352]. In the first study, a PNAGA-based surface was investigated, in which the critical transition temperature could be controlled by means of random copolymerization with the hydrophobic monomer poly(*N*-phenylacrylamide). In the second case, ureido-modified poly(ʟ-ornithine)-*co*-poly(ʟ-citrulline)-based polypeptides were used, whose *T*_c_ could be controlled via the proportion of ureido-modified functionalities. In both systems, a temperature-dependent UCST switching behavior of the surfaces could be detected in terms of changing water contact angles. Furthermore, controlled cell adhesion was verified in both cases using fibroblasts of the cell line NIH-3T3. For the PNAGA system, cells were first incubated for 20 h on the cell adhesive collapsed brush structure at 30 °C (*T* < *T*_c UCST_), which had a θ_SW_ of ≈65° (Figure 11, part B). After applying the temperature trigger, i.e., increasing the temperature to 37 °C (*T* > *T*_c LCST_), ≈94% of the cells can be released due to the conformational change of the brushes to the stretched, hydrophilic state (θ_SW_(*T* = 37 °C) ≈ 30°). Among the released cells ≈98% were still viable. Although, in contrast to LCST systems, the cell release occurs upon increasing the temperature, a cell-adhesive state consistently occurs for collapsed polymer chains (*T* < UCST, *T* > LCST) whereas a cell-repulsive state is present for stretched chains (*T* > UCST, *T* < LCST). However, considering the polypeptide-based UCST system, cell adhesion occurs at high temperatures (*T* = 39 °C; *T* > UCST) in the swollen polymer state, while subsequently about ≈65% of the grown cells were released during the collapse of the thermoresponsive polypeptides upon temperature reduction to 37 °C. The viability of the released cells was similarly high (≈96%) [352]. Despite extensive characterization of the surfaces, the authors were unable to elucidate the fundamental differences in the temperature-dependent cell adhesion of the two UCST systems, although they both rely on nonionic hydrogen bonding. This compelling example demonstrates clearly, that the complex UCST behavior of polymers, especially when anchored to surfaces, is not sufficiently well understood yet. In order to improve this, fundamental mechanistic studies are of particular importance.

Based on preliminary theoretical studies, Murakami et al. investigated the thermoresponse of polystyrene (PS) brushes in cyclohexane [354–355], which is one of the fundamental and well known systems exhibiting UCST behavior. They observed a shift of the binodal line and thus the UCST to lower temperatures of the PS brush system compared to PS chains in solution both in Monte Carlo simulations as well as within experimental data. They attribute this behavior to the spatial restrictions of the polymer chains within the brush structure, which hampers segregation. Interestingly, they also describe in detail a reversible formation of characteristic microdomain structures of the brushes below their critical transition temperature. This unique feature, leading to a structuring of the brush surface, partially resembles the microphase separation of diblock copolymer brushes. Murakami et al. showed that different types of microdomains can be reversibly formed via tuning of the grafting density [354]. While for densely grafted brushes (σ = 0.38 chains/nm^2^) the formation of microdomains is strongly suppressed by the steric restrictions and a homogeneous surface is obtained below *T*_c UCST_ (10 °C), island- (σ = 0.020 chains/nm^2^), bicontinous- (σ = 0.027 chains/nm^2^) and hole-shaped (σ = 0.055 chains/nm^2^) microdomains appear at low grafting densities. While around 20–30 nm large PS-rich islands of segregated polymer chains are formed in a PS poor matrix at σ = 0.020 chains/nm^2^, inversely at higher grafting densities domains of lower PS density (holes) are formed in a PS-rich matrix. In all cases, however, due to the UCST behavior of the brushes, the roughness decreases sharply with increasing temperature and the nanostructuring of the surface disappears above *T*_c UCST_.

Recently, we were able to develop a novel multi-responsive coating with counterion inducible UCST, which we have subjected to extensive mechanistic in situ investigations (Figure 11, part C) [300–301]. Herein, the water-soluble polyelectrolyte PDMAEMA is covalently anchored to a silicon substrate using an effective grafting-to approach. The obtained 5–12 nm thick (dry state) “Guiselin” brushes exhibit LCST behavior in the presence of monovalent salt ions, which is typical for PDMAEMA. However, upon addition of the multivalent ion [Fe(CN)_6_]^3−^, an induced UCST behavior of the brushes was detected. Using spectroscopic in situ ellipsometry, it was possible to monitor this unique temperature-dependent swelling behavior of the brushes in aqueous media for the first time. Moreover, applying in situ ATR-FTIR spectroscopy enabled monitoring of the temperature-dependent electrostatic interactions between the polycationic PDMAEMA brushes and the anionic complex, which govern the molecular mechanism of the induced UCST transition. Due to its polyelectrolyte structure (p*K*_a_ ≈ 7.0– 7.5) PDMAEMA also exhibits responsiveness to both pH and ionic strength. Exploiting the highly charged state of the polycation under acidic conditions enabled shifting of the *T*_c UCST_ to 40.7 ± 2.0 °C at pH 5, whereas a significantly lower *T*_c UCST_ of 34.0 ± 1.2 °C was observed for the brushes at pH 8. Interestingly, under basic conditions this value is significantly higher than in comparable studies of free polymer chains in solution, where a *T*_c UCST_ (at pH 8) of 24 °C was detected via turbidity. Since Plamper et al. [293]. were also able to detect an increase in the induced *T*_c UCST_ for star PDMAEMA compared to linear PDMAEMA, it can be assumed that the steric restrictions of the polymer chains and the resulting high local polymeric segment density in these architectures are responsible for a shift in *T*_c_.

Moreover, by varying the concentration of monovalent NaCl or multivalent [Fe(CN)_6_]^3−^ ions, both of the critical transition temperatures (*T*_c LCST_ and *T*_c UCST_) as well as the switching amplitude of the brushes (change in degree of swelling), the width of the phase transition and its reversibility could be controlled. A linear dependence of *T*_c_ on the logarithmic ionic strength was observed for the LCST transition of the brushes, allowing to adjust the *T*_c_ between 26 °C and 60 °C by increasing the NaCl concentration from 0.001 mM to 100 mM. In addition, a complex pattern of very sharp, jump-like transitions of the brushes at intermediate ionic strength (1–100 mM NaCl) versus a broad transition at low and high concentrations of NaCl were observed, which resembled a theoretical model for thermoresponsive polyelectrolyte microgels [356]. However, for the induced UCST transition of the brushes, an increase in *T*_c_ and particularly sharp phase transitions were observed at high concentrations of the multivalent ion. A maximum film thickness change due to thermoresponsive switching of ≈98% (from ≈24 nm to ≈48 nm) was achieved at a concentration of 100 mM [Fe(CN)_6_]^3−^. Despite all this, however, it is also an extremely sensitive brush where even a low concentration of 0.001 mM multivalent ions leads to a domination of the PDMAEMA inherent LCST behavior and an induced UCST response with an increase in brush thickness with increasing temperature is detected instead.

In addition, applying temperature-dependent in situ AFM revealed fundamental structural differences between UCST and LCST transitions of the nanoscopic coating. Whereas homogeneous surfaces were detected both below and above the *T*_c LCST_ in monovalent salt solutions, pinned PDMAEMA micelles with entrapped multivalent counterions were observed during the induced UCST transition (Figure 11, part C) [301].

While the occurrence of such nanostructures is consistent with recently developed theoretical models [292,357], the in situ study demonstrated for the first time that the characteristic dimensions of the pinned micelles (diameter and height) can be specifically controlled by the superimposed multi-responsive behavior of PDMAEMA towards environmental triggers like temperature or pH value. Moreover, in contrast to the PS-based system of Murakami et al. in which the formation of microdomains solely occurs below the *T*_c UCST_ of the brushes, the structure of pinned micelles persists even above the *T*_c UCST_ due to strong electrostatic interactions with the multivalent ions [354]. The use of grafted UCST-type polymers therefore represents a promising, novel bottom-up strategy to generate and control nanopatterns in aqueous environment, which is an important tool to tailor surface properties of flat substrates but also provides new perspectives for the colloidal stability of grafted nanoparticles. While the effect of multivalent ions on free polyelectrolyte chains has been extensively studied in the past, it was only in 2017 that Lee et al. demonstrated a Ca^2+^-induced UCST behavior of grafted polyelectrolyte brushes on silica particles for the first time [358]. Before going into more detail on grafted particles in the next chapter, we would first like to summarize benefits and limitations of UCST exhibiting brushes on flat substrates (Table 4).

**Table 4:** Benefits and limitations of UCST exhibiting brushes grafted onto the surface of flat substrates.

Benefits	Limitations

• Adaptive surface properties can be obtained in a resource-efficient manner by a nanoscopic polymeric coating. • Often, a shift in *T*_c UCST_ to higher temperatures is observed for ionic UCST polymers in a sterically restrictive brush structure, while the *T*_c UCST_ of non-ionic polymers more closely resembles that of free polymer chains. • Wetting of a surface can be modulated by temperature. UCST based systems demonstrate reproducible and in some cases switching characteristics superior to well-known LCST systems. In addition, the heat required for a UCST transition can usually be provided more easily, than the cooling which is required for LCST based coatings. • Controlled cell adhesion can be obtained with UCST exhibiting coatings. • Anchoring UCST-type polymers to surfaces can lead to adaptive nanostructuring in the form of microdomains, e.g., as pinned micelles in the presence of multivalent ions	• Frequently, a broadening of the UCST phase transition is observed due to the steric constraints of the grafted polymer chains. • A temperature-dependent characterization of grafted polymers is usually more challenging than the analysis of free polymer chains in solution.

### UCST polymers grafted to (nano-)particles

In addition to anchoring UCST-type thermoresponsive polymers on flat substrates, they can also be immobilized on (nano-)particles forming a responsive corona. The functional shell allows to regulate surface properties of the particles in response to environmental triggers, paving the way to novel sensors and drug delivery systems, adaptable lubricants as well as providing colloidal stability or controlled assemblies of nanoparticles [117,359]. Similarly, to the situation at flat substrates, the grafting density and thus the alignment of immobilized polymer chains has a large impact on the thermoresponse of coated nanoparticles. However, the shape of the particle and its curvature additionally define the steric constraints of the grafted polymer chains. Especially for particles with small diameters (≈1– 50 nm), the free volume available to each of the grafted polymer chains is strongly dependent on the curvature of the particle, even considering a constant grafting density on the particle surface. The complex interplay of particle curvature, as well as grafting density and chain length of the grafted polymers determines the local segment density in the particle corona, which significantly determines the thermoresponsive behavior as well as the temperature-dependent colloidal stability of the particles [115]. Due to the large number of available studies in which LCST-type polymers were used to generate responsive nanoparticles, Gibson and O'Reilly, have already been able to critically illuminate some fundamental differences and trends between the thermoresponsive behavior of responsive nanoparticles compared to polymeric brushes on flat substrates or free chains in solution [115]. However, this approach remains somewhat limited due to the lack of experimental studies for polymers with UCST behavior. However, in the following we would like to discuss the still small number of individual works on UCST-based responsive nanoparticles highlighting compelling advantages as well as remaining challenges of these novel systems in order to set the stage for a more systematic, fundamental discussion in the future.

**Figure 12:**
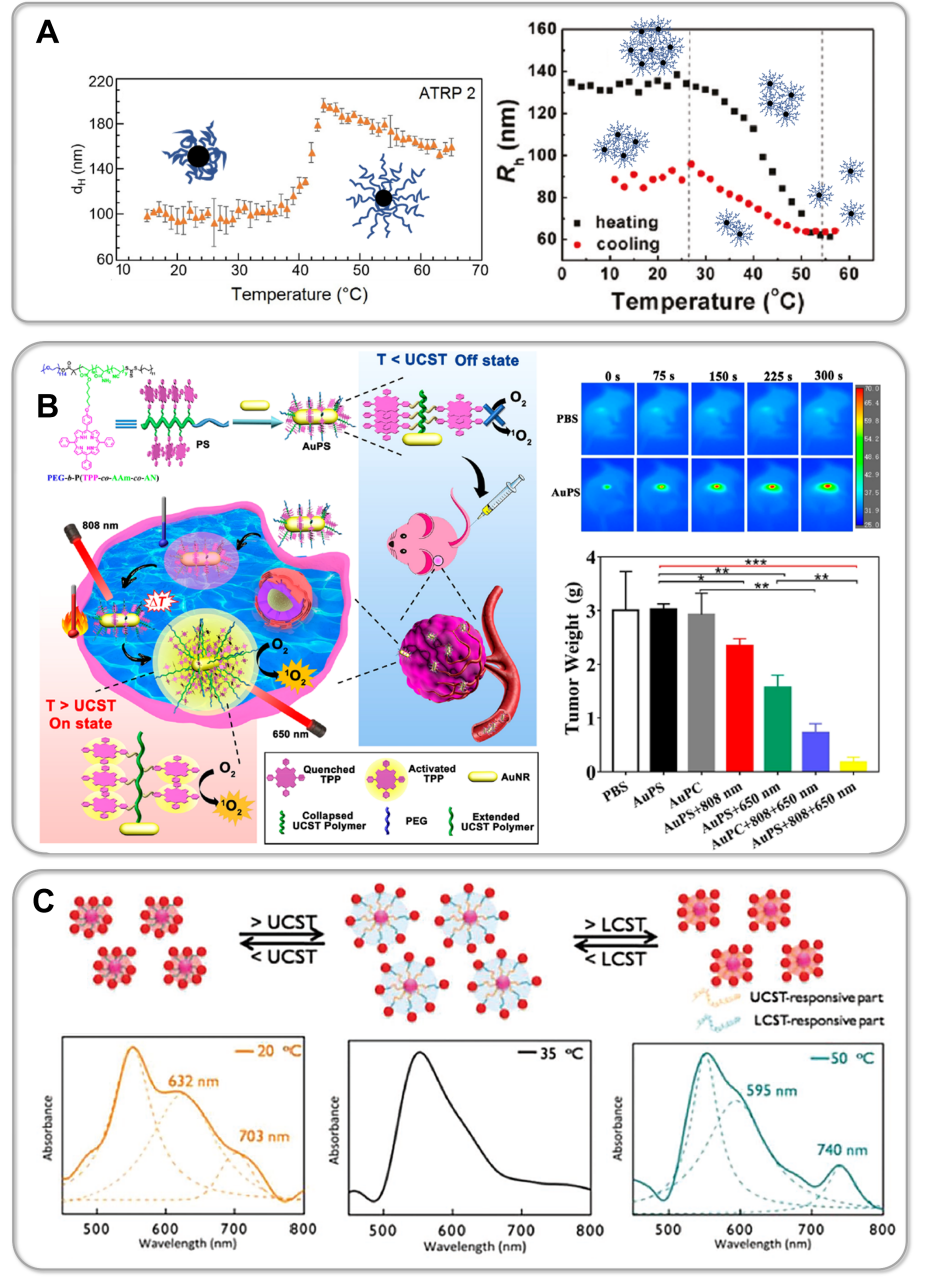
Part A pictures the UCST phase transition of zwitterionic polymers grafted on the surface of mesoporous silica particles. Upon an increase in temperature, this can either lead to an increasing hydrodynamic diameter of the particles due to the swelling of the particle shell as detected by Béltran-Osuna et al. [360] (left) or can proceed via associated states of the particles, resulting in a decreasing hydrodynamic radius as monitored by Dong et. al. [362] (right). Part B demonstrates a hybrid nanomaterial synthesized by Huang et al. [276], which combines an UCST exhibiting copolymer, containing (P(AAm-co-AN), and gold nanorods (left). In vivo NIR radiation of 4T1 tumor-bearing mice leads to plasmonic heating in presence of the nanomaterial (AuPS) in comparison to the injected PBS buffer reference (PBS) as pictured in the IR thermal images (upper right). The subsequently induced conformational transition of the UCST polymer leads to an activation of the photothermal therapy via embedded porphyrin units, which results in a significant reduction of the tumor volume (bottom, right). Part C shows the first core-satellite structure embedding a dual thermoresponsive polymeric linker synthesized by Han et al. in 2018 [365]. The spacing between the gold nanoparticle core and its satellites can be controlled via the thermoresponsive transitions (UCST and LCST), thus leading to a dynamic modulation of the optical properties. Figure 12A (left) was adapted from [360] with the permission of AIP Publishing. © 2020 Á. A. Beltrán-Osuna, J. L. Gómez-Ribelles, J. E. Perilla. Published under license by AIP Publishing. This content is not subject to CC BY 4.0. Figure 12A (right) was adapted with permission from [362]. Copyright 2011 American Chemical Society. This content is not subject to CC BY 4.0. Figure 12B was adapted with permission from [276]. Copyright 2019 American Chemical Society. This content is not subject to CC BY 4.0. Figure 12C was adapted from [365], F. Han et al., “Reversible Thermoresponsive Plasmonic Core-Satellite Nanostructures That Exhibit Both Expansion and Contraction (UCST and LCST)”, Macromol. Rapid Commun., with permission from John Wiley and Sons. Copyright © 2018 WILEY-VCH Verlag GmbH & Co. KGaA, Weinheim. This content is not subject to CC BY 4.0.

In very recent studies, Beltrán-Osuna et al. extensively characterized the thermoresponsive behavior of mesoporous silica particles (95 ± 15 nm particle diameter, 2.8 nm pore size) grafted with zwitterionic poly(sulfobetaine methacrylate) (PSBMA) brushes using surface-initiated ATRP [360–361]. DLS measurements of the dispersed particles in aqueous solution showed a sudden increase of the hydrodynamic radius *D*_h_ upon an increase in temperature, which confirmed the UCST behavior of the responsive particle shell (Figure 12, part A). For all investigated grafting densities (0.16–0.51 chains/nm^2^) as well as molar masses (6500 g/mol ≤ *M*_n_ ≤ 32 000 g/mol) of the zwitterionic brushes this structural transition from a collapsed state at low temperature to a stretched hydrophilic state at high temperature was registered. The largest switching amplitude with an increase from *D_h_* = 109 ± 7 nm for *T* < 40 °C to *D*_h_ = 194 ± 6 nm at *T* ≈ 60 °C, was observed for the polymer brush with the highest molecular weight and the highest grafting density. At high molar masses a broadening of the temperature-dependent phase transition was observed, which is a well-known phenomenon of linear zwitterionic polymers, but occurs there at a considerably higher molecular weight of the polymers [311]. The linear increase of the phase transition temperature with increasing molecular weight of the zwitterionic polymers, which was found here is in good agreement with linear polymers [311]. Beltrán-Osuna et al. found this shifts the *T*_c UCST_ from ≈34 °C for *M_n_* = 6.5 kDa to ≈52 °C for *M_n_* ≈ 32 kDa [360]. This universal dependence is also confirmed for grafted brushes on classical nonporous silica particles [362–363] as well as gold nanoparticles [364], and is also consistent with zwitterionic brushes confined to flat substrates [90]. However, it is interesting to note that, at comparable molecular weight of the polymers, the *T*_c UCST_ for these brush systems is significantly increased compared to free linear chains in solution. Beltrán-Osuna et al. attribute this to the reduction of conformational freedom in these structures, which enhances electrostatic attractions governing the UCST transition [360]. Furthermore, it is pointed out that the local segmental density of the polymer within the brush structure is very high. According to estimates by Durand-Gasselin et al., investigating zwitterionic brushes on gold nanoparticles with similar grafting densities of ≈0.2 chains/nm^2^, a weight fraction of ≈60 wt % polymer can be achieved within the particle corona [364]. Although it has long been known for zwitterionic polymers in solution that the *T*_c UCST_ initially increases significantly in the range up to ≈10 wt % [343], Yu et al. were able to clarify in a recent study that at very high polymer concentrations of 10–40 wt % an extensive decrease of the *T*_c UCST_ can be observed [320]. The authors attribute this to the high viscosity of the solutions and thus limited mobility of the polymer chains, which hinders the formation of attractive polymer interactions essential for UCST behavior. However, in several independent studies it was found that the high local segment density in zwitterionic brushes inversely leads to a strong increase in *T*_c UCST_. Considering different architectures, we conclude that in particular the relative alignment of the polymer chains is crucial for the location of the transition temperature. On the one hand, in zwitterionic brush architectures on flat substrates as well as on particles, the parallel arrangement of the chains often leads to increased ion pair formation and thus increases the *T*_c UCST_ compared to free linear chains. On the other hand, steric constraints in zwitterionic star polymers as well as branched structures or block copolymers usually hamper attractive electrostatic interactions, which leads to a decrease in *T*_c UCST_.

In addition to the shift of the transition temperature, the spatially directed interactions, that govern the thermoresponsiveness of these polymers, can lead to different macroscopic outcomes of the phase transition even in apparently similar architectures. In this context, the colloidal stability of nanoparticles with a thermoresponsive polymeric shell is not only very sensitive to the interplay of particle curvature, grafting density and chain length for grafted LCST polymers, as already discussed in detail by Gibson and O'Reilly, but often leads to contradictory observations for UCST based systems as well [115]. While studies on zwitterionically grafted mesoporous silica [360], polystyrene particles [366] and gold nanoparticles [364], respectively, report an increase in *D*_h_ of dispersed particles during the UCST transition, similar work on classical as well as mesoporous silica particles with a zwitterionic corona show a temperature-dependent assembly behavior that leads oppositely to a decreasing *D*_h_ upon an increase in temperature [362–363,367]. For the first case, Polzer et al. demonstrated via cryo-TEM analysis that the zwitterionic particle corona takes the form of a highly condensed phase at the particle surface supplemented by a diluted layer consisting of individual chains that extend far into the surrounding solution at low temperatures (*T* < UCST) [366]. Nevertheless, even at low temperatures, colloidal stability of the particles is achieved both in water and upon addition of up to 2 mol/L NaCl. Upon heating, a drastic swelling of the responsive particle shell is detected even in the presence of salts. The UCST transition is moreover reproducible and reversible during several heating/cooling cycles without any detectable particle aggregation, which was similarly observed by Beltrán-Osuna et al. [360] and Durand-Gasselin et al. [364]. In contrast, Dong et al. [362–363] and Paramelle et al. [367] show in their work that below the *T*_c UCST_ intrachain associations between the zwitterionic groups lead to particle aggregation and result in the formation of a translucent physical gel (Figure 12, part A). Only by increasing the temperature these attractive associations can be transcended, resulting in an excellently dispersed state of the particles at *T* > *T*_c UCST_. Although in this scenario a comparable *T*_c UCST_ is reported in both the heating and cooling cycle, *D*_h_ does not return to its initial value leading to enhanced aggregation upon multiple temperature cycles. While for LCST-based thermally responsive nanoparticles the grafting density seems to be the key parameter determining the colloidal stability [115], both the grafting density and the molar mass of zwitterionic polymers in the particulate UCST systems summarized here seem to play only a subordinate role. Rather, a sufficiently high particle concentration in solution seems to be decisive for the occurrence of aggregation processes [360]. Moreover, Durand-Gasselin et al. show that aggregation below the *T*_c UCST_ can also be induced by the presence of free polymer chains in solution via a depletion flocculation mechanism [364].

Paramelle et al. show that particles with UCST responsive shells can be exploited for a triggered release of the hydrophilic model compound rhodamine B (RhB) [367]. For this purpose, porous hollow silica nanocapsules modified with zwitterionic brushes were first loaded with RhB at *T* > *T*_c UCST_. Subsequent cooling of the particles in solution leads to a collapse of the brushes at *T* < *T*_c UCST_, which reversibly closes the pores of the particles and efficiently encapsulates RhB. Finally, conjugation of 5 nm gold nanoparticles on the brush surface enables plasmonic heating via laser stimulation (λ = 532 nm), which achieves a photothermally induced burst release of RhB from the capsules during the UCST transition of the zwitterionic particle shell. Interestingly, via plasmonic heating, a rapid release of RhB could be registered within 60 min, while thermal heating resulted in a leakage of RhB up to a time period of 6 h. Hei at el. showed that the robust UCST behavior of P(AAm-*co*-AN) brushes can also be exploited for the controlled release of the anticancer drug DOX from mesoporous silica nanoparticles [368]. By varying the AN content of the copolymer from around ≈7 to 13 mol %, the *T*_c UCST_ could be adjusted between 32–50 °C, similar to micellar carriers. Temperature cycling between 25 °C and 42 °C achieved a step wise release of the loaded DOX from the grafted particles via gating of the porous channels through the conformational changes of the UCST brushes. Moreover, in vitro studies demonstrate the effective uptake of the responsive particles into breast tumor cells and the triggered release of DOX at elevated temperature leading to a reduction in cell viability of more than 20% (42 °C vs 37 °C). A similar burst release of DOX molecules from grafted mesoporous silica particles was recently reported by Hu et al. using the UCST polymer PNAGA [369]. By additionally incorporating a photosensitizer (indocyanine green) into the carrier system, it was possible to trigger the localized generation of heat via NIR irradiation. In analogy to micellar systems, a synergistic effect of this combined chemo- and photothermal therapy could be observed. In a recent work, Amoli-Diva et al. [370] and Huang et al. [276] developed P(AAM-*co*-AN)-based nanocarriers, in which the UCST copolymer was coupled to bimetallic Au-Ag nanoparticles and gold nanorods, respectively. In both cases, laser irradiation and thus excited surface plasmon resonance within the metallic components effectively generated localized heat for the UCST transition of the copolymer. The same approach was successfully applied for responsive hydrogels consisting of (PAAm-*co*-AN) and spherical gold particles or gold nanorods, respectively [371–372]. Here, plasmonic heating represents an advantageous alternative to the use of conventional organic photosensitizers which are often applied in micellar P(AAm-*co*-AN) carriers. Aggregation-induced quenching of the photosensitizer as well as its unselective activation and cytotoxicity can be avoided by plasmonic heating with metallic nanoparticles. The *T*_c UCST_ values of the pure block copolymer P(AAm-*co*-AN) and the nanocarrier systems developed by Amoli-Diva et al. and Huang et al. agree well. The systems show in all cases reproducible and reversible UCST transitions with a slightly lower sensitivity and broader phase transition for the carrier systems. Nevertheless, Amoli-Diva et al. succeeded in reducing the cell viability of breast tumor cells by up to ≈30% via plasmonic heating of the nanocarrier and the triggered release of the anticancer drug letrozole from the P(AAm-*co*-AN) particle corona. Huang et al., on the other hand, exploited an on demand generation of reactive oxygen species (ROS) for targeting tumor cells via the incorporation of porphyrin units into the UCST block copolymer (Figure 12, part B). The collapsed state of the thermoresponsive polymer at *T* < *T*_c UCST_ initially generates a quenched "off" state of the porphyrin units via π–π stacking. After incorporation of the carrier into the tumor tissue, UCST-based stretching of the polymer chains can be obtained via plasmonic heating of the gold nanorods, which subsequently switch the porphyrin units in a ROS-generating “on” state. Both in vitro and in vivo studies show that minimal toxicity of the nanocarrier can be ensured in the “off” state, for example during blood circulation, but also high cell toxicity is achieved in the “on” state after incorporation into breast tumor cells (4T1). In addition to the development of smart carrier systems, UCST polymers are also gaining interest for the controlled self-assembly of nanoparticles. Among other external stimuli, the temperature-responsive self-assembly (TRSA) of polymer-grafted nanoparticles is a particularly promising method, although it has been conducted almost exclusively with LCST exhibiting polymers in the past [373]. Despite of early work on polystyrene brushes grafted from iron oxides nanoparticles, as well as PNAGA grafted onto gold nanoparticles qualitatively demonstrating UCST-based TRSA [374–375], only recently, extensive studies by Tao et al. provide a broader insight in UCST type polymeric ligands for responsive nanoparticles [373]. The utilized ≈21 nm large gold nanoparticles, which were coated with polystyrene ligands, demonstrate a temperature-dependent reversible and reproducible self-assembly into clusters in a water/THF mixture. When the temperature decreased from 36 °C to 21 °C, the particles initially formed small clusters that subsequently grew into larger assemblies, resulting in both a drop in extinction due to partial precipitation as well as a strong red shift of the characteristic localized surface plasmon resonance peak in the UV–vis spectrum. However, upon subsequent increase in temperature, the formed clusters/precipitates were able to dissociate into individual dispersed nanoparticles, thus reversibly modulating their optical properties. Furthermore, by varying the composition of the THF/water mixture the *T*_c UCST_ can be tuned, which provides a second external trigger for controlling the solvent quality and thus the particle assembly. Interestingly, comparison of free PS chains in solution shows that the *T*_c UCST_ of the particles is much lower and, moreover, the particles react much more sensitive to changes in solvent composition. Han et al. successfully fabricated a responsive hybrid core-satellite nanostructure containing an UCST-type polymeric linker for the first time in 2018 (Figure 12, part C) [365]. Well defined nanostructures of this type, though containing LCST-based polymeric linkers between the core and its satellites, have been reported before by Rossner et al. [376] and Han et al. [377]. Unlike these examples the use of UCST polymers not only enables to trigger a single-phase transition, but also, by combination with a LCST-type polymer, enables a dual-thermoresponsive behavior of a core-satellite nanostructure for the first time. The gap distance between the core and its satellites, determining the surface plasmon resonance coupling, can therefore be modulated by two distinct thermoresponsive transitions (UCST and LCST) of the polymeric linker, allowing to tune the optical properties of the assembly dynamically. For this purpose, Han and colleagues synthesized a block copolymer from a zwitterionic betanized PDMAPMA unit with *T*_c UCST_ ≈ 25 °C and a poly(DEGA-*co*-OEGA) segment with a *T*_c UCST_ of ≈45 °C. Similar to what was discussed in detail about double stimuli responsive block copolymers earlier, a shift in the *T*_c UCST_ of the block copolymer is detected in comparison to the UCST of the homopolymer (*T*_c UCST_ betanized PDMAPMA ≈37 °C), while the LCST behavior of the block copolymer strongly resembles that of the incorporated LCST segment (*T*_c LCST_ poly(DEGA-*co*-OEGA) ≈ 45 °C). After successfully embedding the obtained block copolymer in the core-satellite structure composed of gold nanoparticles, DLS measurements demonstrate the dual-thermoresponsiveness of the assembly. While below the UCST at 20 °C a small hydrodynamic radius *D*_h_ of 163.4 ± 3.1 nm is detected due to the collapse of the UCST block within the polymeric linker, an increased *D*_h_ of 174.1 ± 2.7 nm is reported at an elevated temperature of 35 °C (*T* > UCST). Further increasing the temperature to 50 °C (*T* > LCST), which causes the thermoresponsive transition of the LCST segment, results again in a decreased *D*_h_ of 161.2 ± 3.1 nm. The reversible change of the distance between the core and the satellites via temperature cycles successfully controls the plasmonic coupling of the assembly and offers a high potential as a so-called plasmonic ruler, but also for the application as responsive SERS sensor as well as for advanced bioimaging. Finally, we would like to summarize the benefits and limitations of particles grafted with UCST polymers (Table 5).

**Table 5:** Benefits and limitations arising from UCST exhibiting polymers grafted onto the surface of (nano-)particles.

Benefits	Limitations

• Metallic nanoparticles enable plasmonic heating in order to trigger the UCST transition of the polymeric particle shell. Under light irradiation, heat can thus be generated in a localized and temporally controlled manner without the use of typically cytotoxic organic photosensitizers. This approach provides high potential for novel drug delivery systems and sensor applications. • The UCST transition of the particle shell can be used either for a targeted assembly of particles (TRSA) or for providing colloidal stability of particles in a large temperature window. • Within particle assemblies, e.g., gold nanoparticle based core-satellite structures, UCST exhibiting polymeric linkers enable a dynamic modulation of the optical properties via plasmonic coupling.	• Even with supposedly analogous particle architectures, the temperature-dependent colloidal stability or assembly behavior can vary strongly (Figure 12, part A) and is therefore difficult to predict. Because of the small number of available studies, the reasons for these variations are not sufficiently understood until now.

## Conclusion

The present review focused on the correlation of the thermoresponsitivity of polymers with morphological patterns as well as arrangement and conformation of the polymer chains. The consideration included star polymers, polymeric micelles and polymers covalently attached to flat substrates and particles. The focus of our considerations was on polymer systems exhibiting UCST behavior because this temperature-induced phase transition is underrepresented in the literature so far, in contrast to polymers having an LCST. Starting from a basic theoretical consideration and a description of the characteristics of the four included polymer arrangements, the influence of the specific polymeric architectures on the UCST behavior is described. Therefore, the review article provides a deeper understanding of temperature triggered responsive phase transitions that goes beyond previous reviews, which were focused on the description of the macromolecular structure of UCST-type polymers but not on polymer architectures that lead to spatially constrained assemblies. It is demonstrated, that a limited mobility of the polymer chains has a strong impact on the phase transition temperature itself, but also on the sharpness, switching amplitude and reversibility of the UCST transition. Interesting and promising effects, such as amplified secondary triggers in star polymers, inside-out switchable micelles of block copolymers, nanostructured coatings with switchable wettability, as well as the targeted release of drug molecules from grafted nanoparticles via plasmonic heating can be obtained exclusively with these sterically constrained architectures, but not by using free polymer chains in solution. Furthermore, it is pointed out that the temporally and spatially well-controllable supply of heat via irradiation of photosensitizers or plasmonic particles required for UCST-type transitions, offers tremendous advantages over well-known LCST-based systems demanding conventional cooling. Even though interest in UCST polymers continues to grow strongly and more and more novel structures with this characteristic have been synthesized, it is also obvious that the mechanistic understanding of these polymers is still insufficient, often leading to contradictory macroscopic observations even for apparently analogous architectures. More detailed academic studies of UCST-type polymers, both theoretically and experimentally, are required to enable a broad use of these promising polymers in real-life applications as novel sensors, smart coatings, or medical carrier systems.

## Abbreviations

AAm, acrylamide; AFM, atomic force microscopy; AN, acrylonitrile; ATR-FTIR, attenuated total reflection-Fourier-transform infrared spectroscopy; ATRP, atom transfer radical polymerization; CMC, critical micelle concentration; CMP, critical mixing point; DEGA-*co*-OEGA, di(ethylene glycol) ethyl ether acrylate)-*co*-(oligoethylene glycol acrylate); DLS, dynamic light scattering; DNA, deoxyribonucleic acid; DOX, doxorubicin hydrochlorid; EPR, electron paramagnetic resonance; LCST, lower critical solution temperature; GA-polyHMPA, glycolamide-modified poly(*N*-(2-hydroxypropyl)methacrylamide); Gly, glycine; GMA, glycidyl methacrylate; HPG, hyperbranches poly(glycol); IDPs, disordered proteins; LBL, Langmuir–Blodgett layer; NIR, near-infrared; NMR, nuclear magnetic resonance; MEDSAH, [2-(methacryloyloxy)ethyl]dimethyl(3-sulfopropyl)ammonium hydroxide; OEGA, oligoethylene glycol acrylate; PAA, poly(acrylic acid); PAm, poly(acrylamide); PAMPT, poly(3-acrylamidopropyl)trimethylammonium chloride); PBIEM, 2-(2-bromoisobutyryloxy)ethyl methacrylate; PBMA, poly(butylmethacrylate); PBuOX, poly(2-butyl-2-oxazoline); PBOX, poly(2-(3-butinyl)-2-oxazoline); PCL, poly(ε-caporlactone); PCPOX, poly(2-cyclopropyl-2-oxazoline); PDEAAM, poly(*N,N*-diethylacrylamide); PDEAEAM, poly(*N*-(2-diethylamino)ethyl acrylamide)); PDEAEMA, poly(2-(diethylamino)ethyl methacrylate)); PDEGA, poly(di(ethylene glcol)ethyl ether acrylate); PDEGMA, poly(di(ethylene glycol)monoethyl ether methacrylate); PDMAAm, poly(*N,N*-dimethyl acrylamide); PDMAEMA, poly(2-(dimethylamino)ethyl methacrylate); PDMAPMA, poly(*N*,*N*-dimethylaminopropyl methacrylamide); PDMAPS, poly(*N*,*N*′-dimethyl(methacryloylethyl)ammonium propanesulfonate); PEI, polyethyleneimine; PEG, poly(ethylene glycol); PEGMA, poly(ethylene glycol)monomethyl ether)methacrylate; PEtOX, poly(2-ethyl-2-oxazoline); PFOTES, 1*H,*1*H,*2*H*,2*H*-perfluorooctyltriethoxysilane; PHEA, poly(2-hydroxyethyl acrylate); P(HPEI-IBAm), poly(hyperbranched polyethylenimine isobutyramide); PHPMA, poly(2-hydroxypropyl methacrylamide); PLAMA, poly(2-lactobionamidoethyl methacrylate); PLys, poly(ʟ-lysine); PIPOZ, poly(2-isopropyl-2-oxazoline); PMAPMA, poly(2-(*N*-methyl-*N*-(4-pyridyl)amino)ethyl methacrylate); PMDM, poly(2-(2-methoxyethoxy)ethyl methacrylate); PMEMA, poly(2-(*N*-morpholine)ethyl methacrylate)); PMEO_2_MA, poly(di(ethylene oxid) mehyl ether methacrylate); PMeOX, poly(2-methyl-2-oxazoline); PMAA, poly(metacrylic acid); PMMA, poly(methyl methacrylate); PMPC, poly(2-methacryloyloxyethyl phosphorylcholine); PMPEG, poly(monomethoxy poly(ethylene glycol)); PNAGA, poly(*N*-acryloyl glycinamide); PNIPAAm, poly(*N*-isopropyl methacrylamide); PNMA, poly(*N*-methylolacrylamide); PNTBA, poly(*N*-*tert*-butylacrylamide); PNVCL, poly(*N*-vinyl caprolactam); PNVP, poly(*N*-vinyl-2-pyrolidone); POEGMA, poly((ethylene glycol) methyl ether methacrylate); POEOMA, poly(oligo(ethylene oxide)methacylate); poly(AAm-*co*-MDO), poly(acrylamide‐*co*‐2‐methylene‐1,3‐dioxepane); POX, poly(2-oxazoline); PP, polypropylene; PPE, poly(phosphoester); PPO, poly(propylene oxide); PPropOX, poly(2-*n*-propyl-2-oxazoline); Pro, proline; PS poly(styrene); PSBMA, poly(sulfobetaine methacrylate); PSBP, poly(4-((3-methacrylamidopropyl) dimethylammonio) butane-1-sulfonate); PSPP, poly(*N,N*-dimethyl-*N*-(3-(methacrylamido)propyl) ammoniopropane sulfonate); PtBMA, poly(tert-butyl methacrylate); PTEGDA, poly(tetra(ethylene glycol)diacrylate)); PTEGMMA, poly(methoxytri(ethylene glycol) methacrylate); PVAc, poly(vinyl acetate); PVCL, poly(*N*-vinylcaprolactam); PVIm, poly(vinylimidazole); PVME, poly(vinyl methyl ether); P4VP, poly(4-vinylpyridine); RAFT, reversible addition-fragmentation chain transfer; RhB, rhodamine B; ROS, reactive oxygen species; SERS, surface-enhanced Raman spectroscopy; SPIO, supraparamagnetic iron oxide; SPOC, poly(ʟ-ornithin-*co*-ʟ-citrullin); TEM, transmission electron microscopy; THF, tetrahydrofuran; TRSA, temperature-responsive self-assembly; UCNP, rare-earth upconversion nanoparticles; UCST, upper critical solution temperature; UV–vis, ultraviolet-visible spectroscopy; WCA, water contact angle.
